# Mechanisms of action of sacubitril/valsartan on cardiac remodeling: a systems biology approach

**DOI:** 10.1038/s41540-017-0013-4

**Published:** 2017-04-18

**Authors:** Oriol Iborra-Egea, Carolina Gálvez-Montón, Santiago Roura, Isaac Perea-Gil, Cristina Prat-Vidal, Carolina Soler-Botija, Antoni Bayes-Genis

**Affiliations:** 1 ICREC (Heart Failure and Cardiac Regeneration) Research Programme, Health Sciences Research Institute Germans Trias i Pujol (IGTP), Badalona, Spain; 2 CIBER Cardiovascular, Instituto de Salud Carlos III, Madrid, Spain; 3 Center of Regenerative Medicine in Barcelona, Barcelona, Spain; 4 Cardiology Service and Heart Failure Unit, Germans Trias i Pujol University Hospital, Barcelona, Spain; 5 Department of Medicine, Autonomous University of Barcelona (UAB), Barcelona, Spain

## Abstract

Sacubitril/Valsartan, proved superiority over other conventional heart failure management treatments, but its mechanisms of action remains obscure. In this study, we sought to explore the mechanistic details for Sacubitril/Valsartan in heart failure and post-myocardial infarction remodeling, using an in silico, systems biology approach. Myocardial transcriptome obtained in response to myocardial infarction in swine was analyzed to address post-infarction ventricular remodeling. Swine transcriptome hits were mapped to their human equivalents using Reciprocal Best (blast) Hits, Gene Name Correspondence, and InParanoid database. Heart failure remodeling was studied using public data available in gene expression omnibus (accession GSE57345, subseries GSE57338), processed using the GEO2R tool. Using the Therapeutic Performance Mapping System technology, dedicated mathematical models trained to fit a set of molecular criteria, defining both pathologies and including all the information available on Sacubitril/Valsartan, were generated. All relationships incorporated into the biological network were drawn from public resources (including KEGG, REACTOME, INTACT, BIOGRID, and MINT). An artificial neural network analysis revealed that Sacubitril/Valsartan acts synergistically against cardiomyocyte cell death and left ventricular extracellular matrix remodeling via eight principal synergistic nodes. When studying each pathway independently, Valsartan was found to improve cardiac remodeling by inhibiting members of the guanine nucleotide-binding protein family, while Sacubitril attenuated cardiomyocyte cell death, hypertrophy, and impaired myocyte contractility by inhibiting PTEN. The complex molecular mechanisms of action of Sacubitril/Valsartan upon post-myocardial infarction and heart failure cardiac remodeling were delineated using a systems biology approach. Further, this dataset provides pathophysiological rationale for the use of Sacubitril/Valsartan to prevent post-infarct remodeling.

## Cardiology: Heart Failure Medication Reduces Heart Size

The new wonder drug in heart failure management, Sacubitril/Valsartan, rejuvenates the heart by preventing its dilation. Using data from myocardial infarction and heart failure samples, we generated a mathematical model to better understand how Sacubitril/Valsartan modulates pathological heart resize and the combined effect of the drug. Our analysis revealed that Sacubitril/Valsartan mainly acts by blocking both, cell death and the pathological makeover of the outer-membrane of the cardiac cells. These two major processes occur after a heart attack. Most importantly, we discovered a core of 8 proteins that emerge as key players in this process. A better understanding of the mechanism of novel cardiovascular drugs at the most basic level may help decipher future therapies and indications.

## Introduction

Heart failure (HF) is characterized at the myocardial level by ventricular remodeling and dysfunction,^1, 2^ and clinically, by pump failure and sudden death. The principal causes of HF in western countries are coronary artery disease and myocardial infarction (MI).^3^ Important advances have been accomplished in HF management, as the better understanding of neurohormonal activation and agents to block it demonstrated value in improving symptoms and prolonging life expectancy.^4^ Sacubitril/Valsartan (previously known as LCZ696, and marketed by Novartis under the name of Entresto^®^), a novel combination drug, has proven to be superior to conventional angiotensin-converting-enzyme (ACE) inhibition in reducing cardiovascular deaths and HF readmissions, in a large prospective randomized clinical trial.^5^ Given its success, both the American Heart Association/American College of Cardiology and the European Society of Cardiology HF guidelines have rapidly incorporated Sacubitril/Valsartan into their recommendations for HF with reduced left ventricular ejection fraction.^6, 7^ While the mechanism of action for this combination drug is likely to involve the regulation of adverse tissue remodeling, the molecular mechanisms underlying the beneficial effects of Sacubitril/Valsartan (a salt complex at a 1:1 molar ratio),^8, 9^ are, at present, incompletely characterized. Individually, the Sacubitril metabolite LBQ657 inhibits neprilysin, while Valsartan imposes a blockade of the angiotensin II type 1 receptor (AT1R).

All biological processes (e.g., protein–protein interactions or epigenetic regulation) are influenced by their biological context,^10^ with new technologies that fuse engineering and bioinformatics rapidly evolving. Systems biology has arisen as an inter-disciplinary field, based on computational and mathematical models, aimed at unraveling key interactions within complex biological networks.^11–13^ Accordingly, we used in silico systems biology to explore the intricate mechanisms of action (MoA) of Sacubitril/Valsartan as compared to either Sacubitril or Valsartan alone.

To that end, a myocardial transcriptome obtained in response to MI in swine was analyzed to address post-infarction ventricular remodeling.^14^ HF remodeling was studied using public data available in gene expression omnibus (GEO).^15^ A dedicated database and a series of mathematical models, adjusted to known physiological processes, were then used to predict the precise molecular effects of Sacubitril/Valsartan upon the myocardium and vasculature.

## Results

**Fig. 1:**
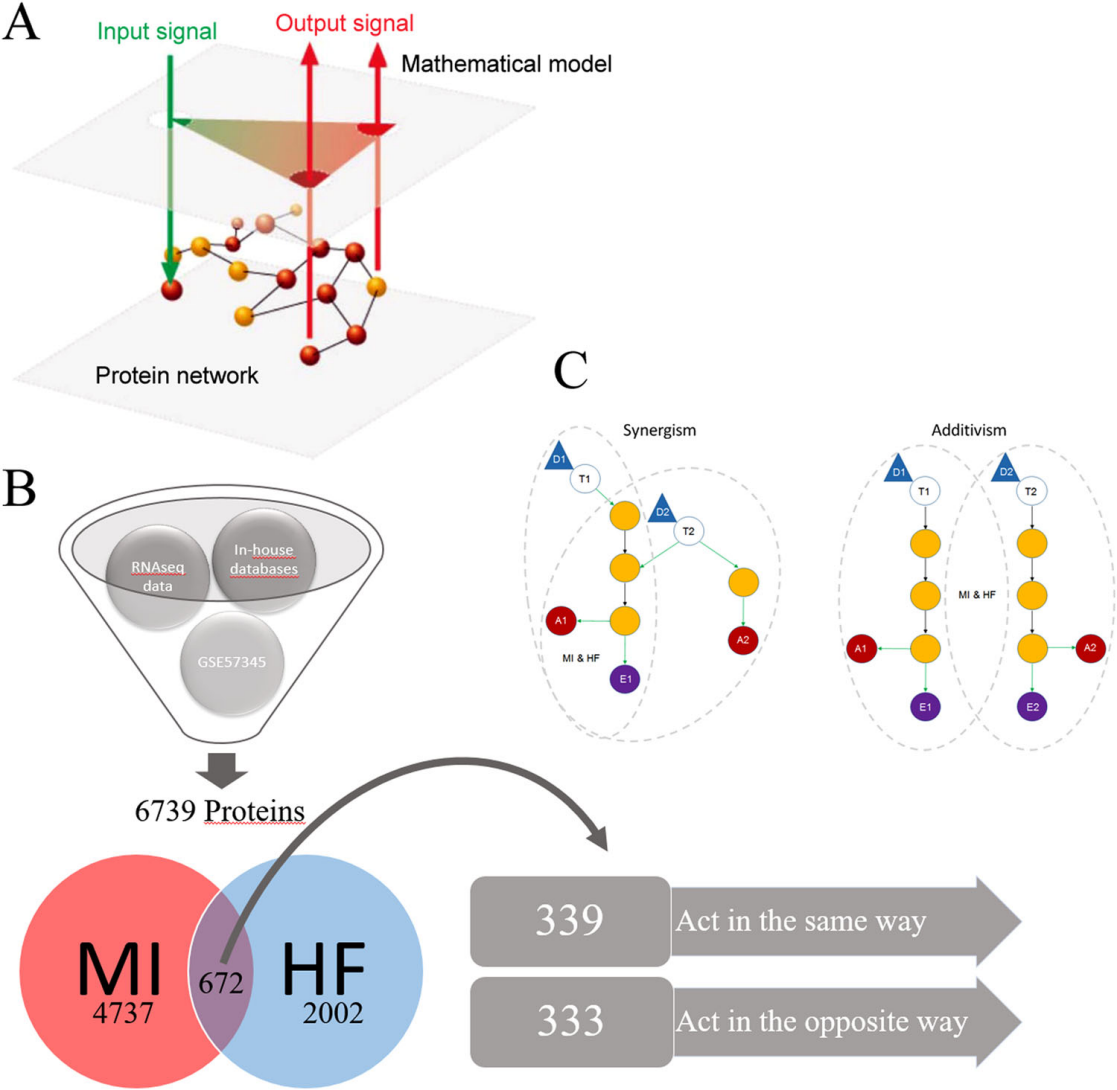
Schematic representation of the mathematical model workflow. **a** Illustration of the input/output data flow. Once all the information is available around Sacubitril/Valsartan targets (eg. Drugbank), the pathophysiology of both conditions (MI and HF) (input signals, *green arrows*) and its links to cardiac remodeling (output signals; *red arrows*) have been identified and characterized at the protein level, the protein network is built. Then the models are trained with all this information and emit how the system is more likely to respond at the protein level (whether by up-regulation or down-regulation) after a certain treatment. Thus, we can elucidate one MoA that is able to explain how the system goes from the stimulus (input) to the observable clinical response (output). **b** Depiction of data processing using TPMS technology. **c** Synergism/additivism schemes. *Triangular shapes* represent drug administration. *Void circles* act as the drug targets. *Yellow circles* represent downstream proteins participating on the cascade. *Red* and *purple circles* refer to different types of effects. *Gray dotted-line* patterns show the pathways of each condition, marking if there is a common share. *MI* myocardial infarction, *HF* Heart Failure

Transcriptome analyses revealed 4737 proteins in the post-infarction cohort (MI), and 2002 proteins comprising the HF disease signature (according to RNAseq data). Collectively, the MI/HF disease signatures shared 672 proteins, of which 339 (50.5%) were directly correlated (e.g. both activated or inhibited in either condition), and 333 (49.5%) exhibited inversely correlated activities (e.g. activated in one condition and inhibited in the other) (Fig. 1b).

### Valsartan and Sacubitril act synergistically to prevent cardiomyocyte cell death and matrix remodeling

An Artificial Neural Network (ANN) was generated to identify the relationships between each drug (Sacubitril, Valsartan, and their combination (e.g., LCZ696)) and the clinical condition under study, e.g., cardiac remodeling. This allowed an assessment of whether Sacubitril/Valsartan acts synergistically or in an additive manner (Fig. 1c). As shown in Table 1, the molecular mechanisms of Valsartan are strongly associated with the prevention of hypertrophy. In contrast, Sacubitril acts by preventing the breakdown of endogenous vasoactive peptides, including natriuretic peptides (ANP, BNP, and CNP), thereby limiting myocardial cell death. In the combination drug, molecular synergy may reverse or reduce left ventricular extracellular matrix remodeling (LVEMR), reduce cardiomyocyte cell death, and, via Valsartan, enhance the effects of Sacubitril. Remarkably, the molecular mechanisms of Sacubitril and Valsartan alone are not associated with LVEMR, and it is only their combination that activates these molecular processes.

**Table 1:** Relationships between the drugs and pathologies under study

Remodeling parameters	Sacubitril	Valsartan	LCZ696	LCZ696 > 120% max (Sacubitril, Valsartan)	LCZ696 > addition (Sacubitril, Valsartan)
Cardiomyocyte cell death	48.00%	15.00%	61.00%	✓	∅
Hypertrophy	31.00%	96.00%	80.00%	∅	∅
Impaired myocyte contractility	5.00%	14.00%	8.00%	∅	∅
LVEMR	21.00%	14.00%	42.00%	✓	✓

The columns Sacubitril, Valsartan, and LCZ696 (Sacubitril/Valsartan), indicate, as a percentage score, the degree of relationship between these drugs and the condition under study; the higher the value, the closer the relationship. The two last columns indicate if the value for their combination exceeds the maximal value achieved by either drug alone with a 20% premium (LCZ696 > 120% max (Sacubitril, Valsartan), or exceeds the sum of both individual drugs (e.g. LCZ696 > addition (Sacubitril, Valsartan). These columns therefore indicate if the combination drug acts synergistically or simply adds the effects of both components.

*LVEMR* left ventricular extracellular matrix remodeling

### Mechanism of action of Sacubitril/Valsartan on myocardial remodeling

**Fig. 2:**
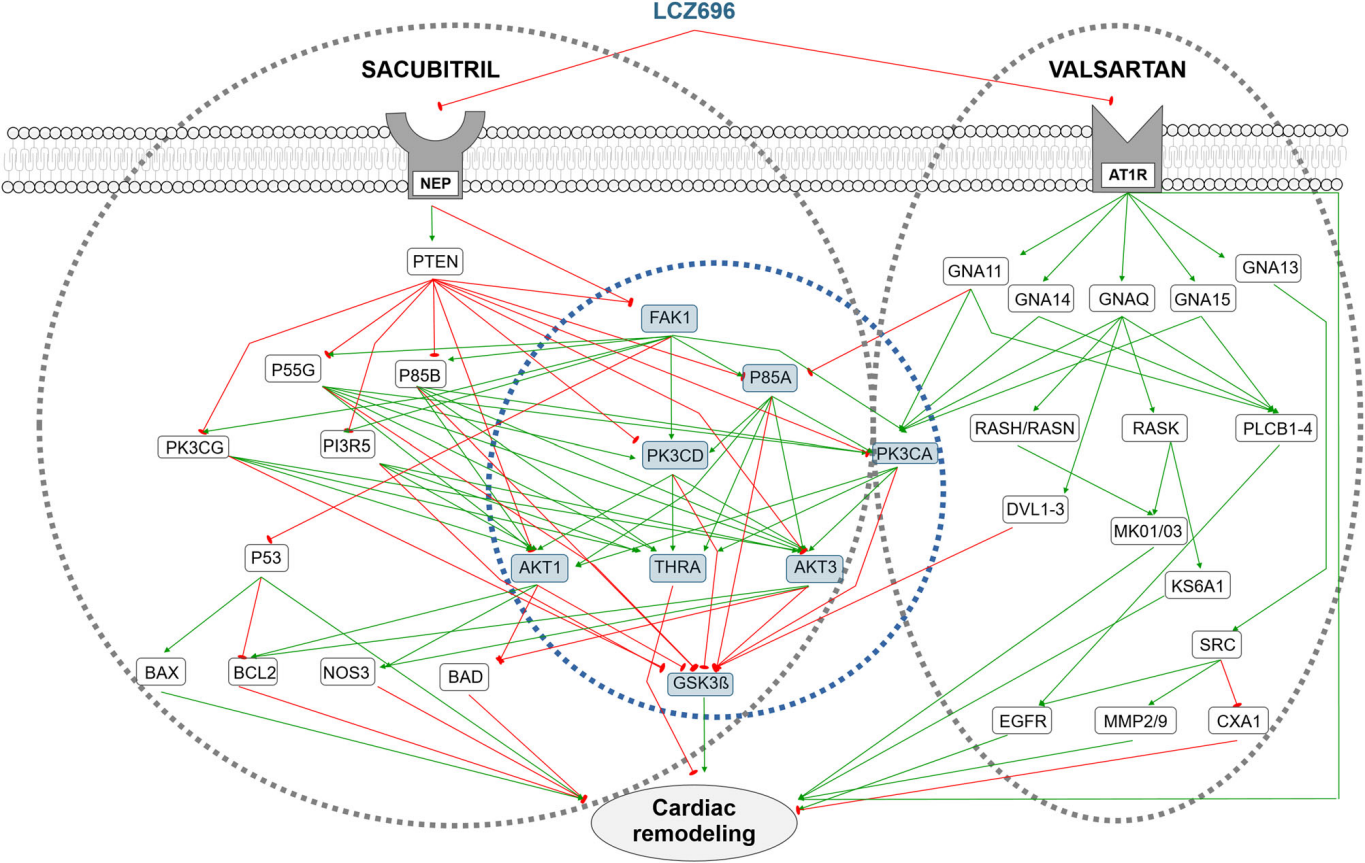
Sacubitril/Valsartan’s MoA. Every relationship depicted represents a mechanism by which the drug could directly or indirectly (via downstream effectors) improve or reverse pathological cardiac remodeling, either through the activation (*green arrows*) or inhibition (*red arrows*) of downstream proteins. The same synergistic MoA was identified for both, MI and HF, cohorts; therefore, the depiction applies for both of them. *Gray dotted-line circles* encompass the proteins affected either by Sacubitril, Valsartan, or both. *Blue dotted-line circle* encompass the core of eight proteins that the mathematical models predict to act synergistically, by being related to both, Sacubitril and Valsartan, pathways in some manner

Based on our Therapeutic Performance Mapping System (TPMS) analyses, the model was able to generate a network displaying the MoA that could explain Sacubitril/Valsartan’s beneficial effects (Fig. 2). The same synergistic MoA was identified in the two cohorts, post-MI and HF, with many pathways corroborating the notion that Valsartan potentiates the effects of Sacubitril.

The MoA included 46 proteins, 8 acting synergistically (Supplementary Table 5), with 18 protein effectors of cardiac remodeling: 6 involved in hypertrophy, 4 in LVEMR, 6 in cardiomyocyte cell death, and 2 in impaired myocyte contractility (Table 2). The MoA contains 23 proteins from microarray data derived from MI, and 10 from HF; 17 of the 23 proteins linked to MI are modulated by the administration of Sacubitril/Valsartan in an opposite fashion to that induced by infarction (as indicated by transcriptome data), indicating the drug’s beneficial effects. In the case of HF restriction, this effect was observed in 8 of 10 proteins.

**Table 2:** Protein effectors of cardiac remodeling included in the MoA representation

Uniprot	Displayed Name	Protein name	Synergistic node	Remodeling effector
P00533	EGFR	Epidermal growth factor receptor	✗	Hypertrophy
P09038	FGF2	Fibroblast growth factor 2	✓	Hypertrophy
P28482	MAPK1	Mitogen-activated protein kinase 1	✗	Hypertrophy
P27361	MAPK3	Mitogen-activated protein kinase 3	✗	Hypertrophy
Q15418	RPS6KA1	Ribosomal protein S6 kinase alpha-1	✗	Hypertrophy
P30556	AGTR1	Type-1 angiotensin II receptor	✗	Hypertrophy
P17302	GJA1	Gap junction alpha-1 protein	✗	Left ventricle extracellular matrix remodeling
P08253	MMP2	72 kDa type IV collagenase	✗	Left ventricle extracellular matrix remodeling
P14780	MMP9	Matrix metalloproteinase-9	✓	Left ventricle extracellular matrix remodeling
P01137	TGFB1	Transforming growth factor beta-1	✓	Left ventricle extracellular matrix remodeling
Q13490	BIRC2	Baculoviral IAP repeat-containing protein 2	✓	Cardiomyocyte cell death
Q92934	BAD	Bcl2-associated agonist of cell death	✗	Cardiomyocyte cell death
P04637	TP53	Cellular tumor antigen p53	✗	Cardiomyocyte cell death
P49841	GSK3B	Glycogen synthase kinase-3 beta	✓	Cardiomyocyte cell death
Q07812	BAX	Apoptosis regulator	✗	Cardiomyocyte cell death
P10415	BCL2	Apoptosis regulator	✗	Cardiomyocyte cell death
P29474	NOS3	Nitric oxide synthase, endothelial	✗	Impaired myocyte contractility
P10827	THRA	Thyroid hormone receptor alpha	✓	Impaired myocyte contractility

### Valsartan improves cardiac remodeling by inhibiting guanine nucleotide-binding proteins

The inhibition of AT1R seems to inactivate or reduce the activity of a series of cascades that participate in cardiac remodeling, via inhibition of different subunits of guanine nucleotide-binding proteins. For example, inhibition of guanine nucleotide-binding protein G(q) subunit alpha (GNAQ) blocks the ERK1/2 pathway, and the ribosomal protein S6 kinase alpha-1 (KS6A1) (Fig. 2). These are both effectors of cardiac hypertrophy through their inhibition of Ras GTPase superfamily members (RASH, RASN, and KRAS) (Supplementary Fig. 5). The model indicates that ERK signaling is also a potential synergistic pathway for Sacubitril/Valsartan’s effects on cardiac remodeling, possibly acting via FAK1, downstream of Sacubitril binding (Supplementary Fig. 6).

Valsartan also contributes to the synergistic effects mediated downstream of Sacubitril via its regulation of glycogen synthase kinase-3 beta (GSK3B) and the activities of the segment polarity protein disheveled homologs DVL-1, 2, and 3 (Fig. 2) (Supplementary Fig. 5).

Inhibition of the guanine nucleotide-binding protein subunit alpha-13 (GNA13) induces the inactivation or attenuation of the activity of the proto-oncogene Scr kinase, thus producing a reduction of LVEMR through inhibition of matrix metalloproteases-2 and 9 (MMP-2 and MMP-9), and gap junction alpha-1 protein (CXA1). At the same time, a reduction of hypertrophy is achieved through inhibition of the epidermal growth factor receptor (EGFR) (Supplementary Fig. 5). Inhibition of guanine nucleotide-binding protein subunit alpha-11, 14, and 15 (GNA-11,14,15), along with GNAQ, inhibits 1-phosphatidylinositol 4,5-bisphosphate phosphodiesterase beta-1, 2, 3, and 4, thus reducing cardiac hypertrophy through EGFR blockade.

Additionally, inhibition of GNA11 is one of Valsartan’s pathways involved in the synergistic effects of Sacubitril/Valsartan. This is achieved through activation of phosphatidylinositol 3-kinase regulatory subunit alpha (P85A), and phosphatidylinositol 4,5-bisphosphate 3-kinase catalytic subunit alpha isoform (PK3CA), whose activities affect different pathways involved in cardiomyocyte cell death, hypertrophy, and impaired myocyte contractility.

### Sacubitril attenuates cardiomyocyte cell death, hypertrophy, and impaired myocyte contractility by inhibiting PTEN

The inhibition of PTEN mediated by Sacubitril’s effect on neprilysin appears to be the initiator of a series of cascades that participate in cardiac remodeling by inducing the activation of different potential synergistic nodes (Fig. 2). Sacubitril’s downstream effects include attenuation or inhibition of cardiomyocyte cell death through activation of p53, which regulates the activity of Bcl2 and Bax. It also helps to reduce hypertrophy and enhance myocyte contractility through the activation of AKT1 and AKT3, which inhibit the endothelial nitric oxide synthase (NOS3), and activation of the thyroid hormone receptor alpha (THRA) (Supplementary Fig. 7).

### Specific molecular mechanisms of Sacubitril/Valsartan in reducing myocardial remodeling in MI

Interestingly, although our model indicates that the MoA network is the same between MI and HF, specific proteins associated only with the efficacy of Sacubitril/Valsartan for MI patients have been identified (Supplementary Table 6). These include fractalkine, involved in angiogenesis, wound healing, inflammatory processes, and responses to hypoxic conditioning.^16, 17^ The C-type lectin domain family 7 member A (CLEC7A), is necessary for TLR2-mediated activation of NF-Κβ, mediates inflammatory processes, the production of reactive oxygen species, and plays a role in carbohydrate mediated signaling.^18–21^ Urokinase plasminogen activator surface receptor is involved in blood coagulation, apoptosis and cellular metabolism.^22–25^

Although MYH6 was included as a biological determinant in the HF but not infarction model (according to microarray data), this protein ultimately does not seem to participate in Sacubitril/Valsartan efficacy in HF, but may play a role in MI instead. In the case of MYH7, this protein was not included as a determinant in any model, but is involved in the efficacy of Sacubitril/Valsartan’s action in both the MI and HF models.^26, 27^

### Gene ontology and enrichment analyses pinpoint potentially relevant pathways affected by Sacubitril/Valsartan

An enrichment analysis of the proteins shared by the Valsartan and Sacubitril networks identified additional pathways that could be involved in their synergistic effects (Supplementary table 7). As a first approach, we focused our analyses on those pathways where all the relevant activities were present in that set of shared proteins. This approach revealed that the regulation of free fatty acids that modulate insulin secretion, the orexinergic system (Orexin and neuropeptides FF and QRFP), and angiotensin metabolism (target pathway of Valsartan), are the most enriched pathways. Then, we expanded our analyses by focusing on the most relevant pathways according to *p*-value. This approach added, GPCR downstream signaling, the gastrin-CREB signaling pathway via PKC and MAPK, plasma membrane estrogen receptor signaling, PAR1 and PAR4-mediated thrombin signaling events, and calcium signaling and platelet activation. Interestingly, the ACE inhibition pathway was also identified. The P2Y receptors pathway, which could constitute a therapeutic target with which to regulate cardiac remodeling and post-ischemic revascularization,^28^ was also enriched.

To complement this study, a gene ontology analysis was then performed to map our list of shared proteins and pathways in MI and HF to biological processes, allowing us to generate a detailed map of the potential MoA of Sacubitril/Valsartan. This analysis confirmed the GPCR signaling pathway to be the most relevant, according to *p*-value, and displayed processes related to blood circulation, the regulation of systemic arterial blood pressure, and wound healing as highly affected.

## Discussion

**Fig. 3:**
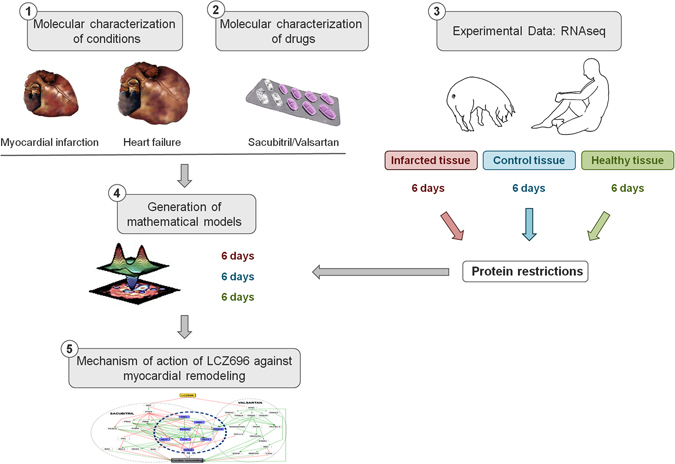
Depiction of the systems biology approach used to identify the mechanism of action of Sacubitril/Valsartan. At the *top left corner*, number 1 and number 2 depict the molecular characterization of both pathologies, MI and HF, and the drug under study, Sacubitril/Valsartan. Obtained from data mining, will serve to generate a truth table that every mathematical model must satisfy. At the *top right corner*, number 3 shows the recollection of experimental data to generate a pathology signature for MI and HF (original pool of proteins). After analyzing all of them, only those previously related to cardiac remodeling and present in both sets of proteins (MI and HF) were used for the study (136 unique proteins), serving as protein restrictions. Number 4 depicts the generation of mathematical models using all the data collected in previous steps 1–3. Finally, number 5 displays the graphical representation of the Mechanism of Action (MoA) found by the mathematical models. All these steps, excluding the experimental data generation, explain how TPMS technology works. LCZ696 denotes Sacubitril/Valsartan

This is the first study to deepen our understanding of the molecular MoA of Sacubitril/Valsartan in MI and HF. Since its development and commercialization, many studies have assessed its pathophysiological effects, as well as pharmacokinetics and pharmacodynamics. However, relatively few studies have attempted to decipher the underlying signaling pathways responsible for its beneficial effects. Using a systems biology approach, we identified the main pathways regulated by Sacubitril/Valsartan implicated in reverse cardiac remodeling (Fig. 3). In previous work, our group identified myocardial gene expression patterns in response to MI in swine.^14^ Using these transcriptome data, together with a public HF cohort (public data available in GEO, accession GSE57345, subseries GSE57338),^15^ we generated a series of mathematical models with which to predict the molecular outcomes of administering Sacubitril/Valsartan. The models do not provide a single solution, but rather identify a universe of possible solutions, where the population of solutions accounts for the variety of physiological responses that may occur in human populations. This is consistent with nature, where we find different molecular responses to the same stimulus (e.g., different side effects observed in individuals treated with the same drug) and different mechanistic explanations to the same biological response (e.g., multifactorial diseases).

In this study, we analyzed the mechanism of action of Sacubitril and Valsartan independently, as well as in combination as the salt complex, always regarding adverse cardiac remodeling. In this case, Fig. 2 represents the MoA that more solutions comply with, and displays the molecular mechanisms most likely to underpin the synergistic action of the combination drug. Not all genes or proteins calculated to be involved in the MoA of each individual solution are represented; only those most mathematically relevant. All the pathways depicted explain how Sacubitril/Valsartan helps to overcome pathological cardiac remodeling, whether by activation or inhibition of certain key proteins. The schematic shows the eight protein nodes most highly implicated in its synergistic action, with an additional six nodes (Supplementary Table 5) that, although not constituting primary hits, may also play an important role.

Our analyses found that Sacubitril/Valsartan modulates cardiac remodeling, acting upon hypertrophic processes, via Valsartan, and limiting myocardial cell death, via Sacubitril. Indeed, it has recently been reported that Sacubitril/Valsartan has the potential to lower high-sensitivity troponin T (hs-TnT) levels, a biomarker for myocyte injury and myocardial cell death, in HF patients.^29, 30^ Taking this into account, our analyses now provide a new perspective on relevant, though previously unknown, MoA.

Moreover, the data presented here reveal that, when combined, Sacubitril/Valsartan act synergistically by reducing LVEMR. This potential relationship had been suggested before, but without a supporting molecular mechanism.^31^ HF patients are prone to suffer sudden cardiac death, more so in ischemic cardiomyopathy due to large myocardial scarring.^32^ It is well known that extracellular matrix remodeling and fibrosis promote lethal arrhythmias.^33^ Remarkably, patients treated with Sacubitril/Valsartan experienced a reduced risk of this condition.^31, 34^ Our in-silico data allow us to speculate that this beneficial effect may, at least in part, be due to the drug combination acting to reduce LVEMR.

The comparison between the mechanism of action of Sacubitril/Valsartan in MI and HF did not show major differences between the two pathologies, but a more in depth investigation revealed 6 proteins that were specifically related to the MI signature alone (Supplementary Table 6). Even though both conditions share many common pathways, MI and HF have different characteristics and impose distinct cardiac environments. This is highlighted by the fact that the expression of 50% of their protein targets is inversely correlated (e.g., expressed in the opposite fashion in either condition (Fig. 1b)). Our analysis was able to pinpoint key differential regulators between both pathologies, and may help to highlight new targets with which to focus future treatments.

The analysis displays the mechanism most likely to be affected according to our methods, which in this case turned out to be intracellular signaling. This was found to be the most robust pathway affected, but surely it can be that this is not the only one. The pharmacological effect of Sacubitril/Valsartan on cardiac remodeling could encompass, and most certainly be affecting, cell to cell communications as well as a wide variety of physiological cardiac phenotypes, such as the decrease in blood pressure, changes in heart rate, mechanical stress, valvular disorder, etc. However, our models take into account all of the proteins involved in cardiac remodeling present in our cohorts (regarding many different pathways, including those outlined above), and all the possible relationships between them (according to the current literature). Doing so, we can ensure that the models are representative and not biased towards a specific pathway.

In vivo studies are currently being designed to characterize ventricular remodeling using novel imaging techniques in patients with HF and reduced ejection fraction. An ongoing study intends to measure the effects of Sacubitril/Valsartan compared to baseline standard medical HF therapy on reverse remodeling using echocardiographic endocardial surface analysis techniques to assess changes in ventricular volume, function, and shape. Metaiodobenzylguanidine scintigraphy and the heart to mediastinum ratio will also be used to assess left ventricle volume regression and risk reduction (NCT02754518, see https://clinicaltrials.gov/ct2/show/NCT02754518 for details). Also, in the setting of MI, in vivo studies are being evaluated. The PARADISE-MI Trial is testing the hypothesis that Sacubitril/Valsartan can reduce cardiovascular death, HF hospitalizations, and the new onset of HF in patients at high risk for ventricular remodeling and HF after MI.

We would like to emphasize that this MoA is not the complete mechanism by which the drug combination could reduce myocardial remodeling after a MI, or reverse myocardial remodeling after HF, but instead highlights the synergy achieved by the combination therapy.

The MoA provided by the model is validated in a two-step process. First, we checked that each link was accurate, e.g., was already described in the literature. Second, we checked that the MoA made sense overall, featuring pathways coherent with the living system, the combinations of drugs assessed (Valsartan and Sacubitril) and the known pathophysiology of cardiac remodeling.

Furthermore, synergistic MoAs are often so complex, and involve such a bewildering number of molecules, proteins and/or genes, that a complete representation in one schematic is unfeasible.

In the last few years, high-throughput technologies have generated an incredibly large amount of data across all omics’ fields. Combined with the rapidly growing field of bioinformatics, these resources provide the scientific community with the opportunity to analyze and test hypothesis with a wider angle of view. In this regard, where researchers can not focus and study every single possibility, due to their extensiveness, model-based approaches and network analyses stand as potent and helpful tools. Moreover, as they integrate so much data, usually are able to provide insights and find relationships that could go easily overlooked with other approaches. As demonstrated in our study, these models are able to generate new data-driven predictions, which then researchers can put to validation and thus expand our capability to tackle complex biological frames that are otherwise inaccessible.

## Conclusions

Using a systems biology approach, we delineated those molecular mechanisms of Sacubitril/Valsartan most likely to attenuate ventricular remodeling. When analyzed independently, Sacubitril was found to attenuate cardiomyocyte cell death, hypertrophy, and impaired myocyte contractility by inhibiting PTEN, thus triggering a series of cascades that participate in cardiac remodeling. On the other hand, Valsartan improves cardiac remodeling by inhibiting the guanine nucleotide-binding protein family. More importantly, our study found that the combination of Sacubitril and Valsartan acts synergistically against LVEMR and cardiomyocyte cell death, with Valsartan enhancing the effects of Sacubitril. By generating an ANN, we were able to create a network displaying the MoA of Sacubitril/Valsartan, and pinpoint the key synergistic nodes associated with its beneficial effects.

## Methods

### Transcriptome database

Transcriptomic data were derived from two well-characterized cohorts.^14, 15^ On one hand, we used microarray data from myocardial gene-expression patterns 1-week after infarct induction in a swine model to explore post-infarct myocardial remodeling. First, data were filtered to discard all entries with inconsistent or contradictory information (e.g., two entries for the same gene name with negative and positive ratio values, respectively), and to identify the number of unique genes altered. Next, swine transcriptomics were translated to their human equivalents via Reciprocal Best Hits (RBH) with BLAST and Gene Name Correspondence. The InParanoid database^35^ was used to identify pig-to-human reciprocal best hits. In the case RBH has not been found for a protein, the reviewed UniProt entry for human protein with a matching gene name was used as a correspondence. The proteins then are tagged with a certain “state” on basal conditions (what we find in healthy normal conditions), whether a protein is activated or inhibited (defined by False Discovery Rate (FDR) at a 0.01 and logFC > 0.25), and we use this information as reference to restrict detections to variability from these values. After discarding contradictory and duplicate entries, with translation to the human proteome, proteins with a human UniProt IDs within each cohort were used as restrictions (restrictive criteria) for our models (Supplementary Fig. 1).

An extra RNA sequencing (RNA seq) dataset (public data available in GEO, accession GSE57345, subseries GSE57338), from failing and non-failing hearts, was included to explore HF ventricular remodeling. Data were processed using the GEO2R tool.^36^ Data derived from both, gene expression microarrays and RNA seq, were compiled and processed by means of the neqc method for normalization,^37^ and Linear Models for Microarray Analysis.^38, 39^ Both allow the identification of differential expression and calculate fold‐change (FC); *p*‐values obtained for each probe were adjusted using the Benjamini–Hochberg FDR at a 0.01 significance level.^40, 41^ Only genes with an adjusted *p*-value < 0.01, and logFC > 0.25 were considered (same threshold applied to swine transcriptomic data). Gene information was one-to-one mapped to proteins for introduction into the protein network.

### Molecular characterizations of pathology and drug

Via manual curation of the literature, we identified the relevant pathophysiological processes implicated in cardiac remodeling and the described interactions of Sacubitril/Valsartan, which were, then, further characterized at the protein level. Thus, we defined a set of pathophysiological restrictions defining cardiac remodeling (all the intrinsic characteristics of the pathologies causing adverse cardiac remodeling, which the mathematical models have to comply with). We included all the proteins (136 unique) (Supplementary Table 1) reported to play a role in cardiac remodeling. These proteins were used to focus our analyses on the pathological conditions of interest in the human biological network. These processes were either implicated as being causal, or a consequence of pathology, with differential classifications possible according to MI or HF. From these proteins, we built a protein network and the mathematical model.

#### Manual curation of the literature

We conducted an extensive and careful review of full-length articles in the PubMed database that included the following search strings (Supplementary table 2): ‘heart failure’(HF) AND’MI’AND ‘pathology’OR ‘physiology’, ‘heart’AND ‘cardiac remodeling’, ‘Angiotensin II’AND ‘neprilysin’AND ‘entresto’, ‘Sacubitril’AND ‘Valsartan’AND ‘HF’AND’MI’AND ‘dysfunction’AND ‘remodeling’, ‘HF’AND’MI’AND ‘stress’.

The search was expanded using the ‘related articles’ function and article reference lists. Only English-language articles were included.

### TPMS technology

TPMS^42–46^ uses as input a combination of biological information drawn from manual curation of the literature and public and private databases (e.g., Reactome, MINT, BioGrid) and experimental information about the disease under study. Then, TPMS creates mathematical models of the patients (either real, when using clinical data, or objective of treatment/study, when using preclinical data), which displays the mechanistically-based result that explain the observable clinical outcomes, in this case adverse cardiac remodeling (output). A simple example of input–output pair would be drug-indication, as for example acetylsalicylic acid, and headache; both clinical terms are then translated to the protein level (i.e., acetylsalicylic acid’s molecular target, and the whole set of proteins and genes whose function modulations have been associated with headache).Typical results of TPMS are: a compound’s MoA, a molecular mechanism of a gene/protein function modulation or combinations thereof, repositioning of compound, finding of therapeutic targets for a given disease, mechanistically-rooted biomarkers, clinical prediction of the efficacy and safety of a compound, etc.

#### Generation of mathematical models

The mathematical models were constructed through TPMS technology. Through the use of artificial intelligence and pattern recognition techniques (based on optimization methods of genetic algorithms (GA), where GAs differ from traditional methods by working with a coding of the parameter set (instead of the parameters themselves), searching from a population of points, using payoff (objective function), and probabilistic transition rules),^47–49^ this technology generates mathematical models that integrate all the available biological, pharmacological and medical knowledge and are able to suggest mechanistic hypotheses that are consistent with actual biological processes, e.g. to simulate human physiology in silico. This goal is achieved by compiling information about the drug (Sacubitril/Valsartan) and/or the disease under study (post-MI and HF ventricular remodeling), and then incorporating it into the biological effectors database (BED).^42, 43^ TPMS BED, is a hand-curated database that relates biological processes (adverse drug reactions, indications, diseases and molecular pathways) to their molecular effectors, i.e. each one of the proteins involved in the physiological process (Supplementary Fig. 2). To train the mathematical models, a collection of known input-output physiological signals was used (Fig. 1a), these being obtained from literature mining and a compendium of databases that accumulates biological and clinical data^50–55^ (Supplementary table 3 and Supplementary table 4). This collection of known input‐output physiological signals generates a list of physiological rules or principles applied to all humans or particular pathophysiological conditions (e.g., known data about targets, MoA of drugs, and their clinical observable effects). These set of rules, the “truths”, are collated to form a truth table that every constructed mathematical model must satisfy. The information contained in the truth table is then used to model complex relationships between inputs and outputs or to find patterns in data.^56–58^ Transcriptomic data were then compiled, analyzed, and imported into the models, having met the reliability criteria outlined above.

The models are able to weight the relative value of each protein (node). However, the large number of links exponentially increases the number of parameters that have to be solved. Different approaches and optimization systems can be called upon in this scenario. These may be based on randomized systems (such as a Montecarlo based system),^59^ or use information derived from the topology of the network.

#### Solving the mathematical models

TPMS technology includes two different and complementary strategies to solve mathematical models:

ANNs: ANNs are supervised algorithms, which identify relations between drug targets and clinical elements of the network.^43, 60^ This strategy is able to identify relationships among regions of the network by inferring the probability of the existence of a specific relationship between two or more protein sets (relationship between Sacubitril/Valsartan protein targets and cardiac remodeling pathway), based on a validation of the predictive capacity of the model towards the truth table. The creation, validation, refinement and checking of the mathematical model that explains the behavior of the network is done by using known data (Known Input) about targets, MoA of drugs (Hidden MoA), and their clinical observable effects contained in truth table (Known Output) (Supplementary Fig. 3).

The raw information that is fed into the network is known as Input layer. The learning methodology used consisted in an architecture of stratified ensembles of neural networks as a model, trained with a gradient descent algorithm to approximate the values of the given truth table. In order to correctly predict the effect of a drug independently of the number of targets, different ensemble of neural networks are trained for different subset of drugs according to their number of targets (drugs with 1 target, 2 targets, 3 targets…). Then, the predictions for a query drug are calculated by all the ensembles, and pondered according to the number of targets of the query drug.

Specifically, the neural network model used is a multilayer perceptron (MLP) neural network classifier.^61–63^ MLP gradient descent training depends on randomization initialization. In this way each training process, applying exactly the same truth table, can give slightly different resulting models. In order to generate each of the ensembles, 1000 MLPs are trained with the training subset. The best 100 ones are used as ensemble. When a new drug-indication pair has to be classified as probable or false, the features describing the topological relation between targets and indication effectors are classified with each of the ensembles; in order to obtain the most accurate prediction, the difference between the number of targets of the query (number of targets of Sacubitril and/or Valsartan) and the number of targets of the drugs used to calculate each ensemble is used to ponder the result of each ensemble. The higher the difference between the numbers of targets, the less weight the results for this ensemble of neural networks have in the final prediction calculation. The output is one node which corresponds to the relationship between a certain drug and its adverse effect (AEs) or indication (Yes-1 or No-0).

Sampling methods: This second strategy is used to describe all plausible relationship between sets of proteins previously identified with ANNs as suggested by experimental work, where each parameter corresponds to the relative weight of a link, connecting nodes (genes/proteins) in a graph (protein map). Thus, this approach does not provide a single solution, but rather identifies a universe of possible solutions that satisfy the biological restrictions of the truth table. However, not all solutions are used for the analysis. The accuracy is calculated by checking how much the models comply with the truth table, and it is defined as the percentage of true positives (correct predictions respect the knowledge stored in the truth table) of the mathematical solution respect the total of parameters to evaluate. The solutions used in subsequent analysis present accuracy higher than 95%. That is, only MoAs that are plausible from the standpoint of currently accepted scientific understanding were considered in the analysis. Once a response (in this case cardiac remodeling) is identified to a specific stimulus (Valsartan and/or Sacubitril), it is possible to analyze the molecular mechanisms that justify this association using the sampling methods strategy (Supplementary Fig. 4). Through this methodology, TPMS technology generates models that comply with the biological restrictions of the truth table. By tracing the changes occurred in the model after applying known pairs of stimulus-response signals, we are able to assess how perturbations are transmitted across the network, thereby adding a dynamic component to an otherwise static model.

## Electronic supplementary material

Supplementary Tables

Supplementary Figure Legends

Supplementary Figure 1

Supplementary Figure 2

Supplementary Figure 3

Supplementary Figure 4

Supplementary Figure 5

Supplementary Figure 6

Supplementary Figure 7
