# Supplementary material for: Mechanisms of action of sacubitril/valsartan on cardiac remodeling: a systems biology approach
Source: NPJ Syst Biol Appl. 2017 Apr 18;3:12. doi: 10.1038/s41540-017-0013-4 (PMC5460292; doi:10.1038/s41540-017-0013-4)
Supplement: Supplementary file 1 — Supplementary Tables [file 41540_2017_13_MOESM1_ESM.docx]

**Supplementary Table 1:** **List of proteins for cardiac remodeling used to focus our analyses on the pathological condition of interest.** Motive ID refers to the remodeling condition that the protein is associated with; 1: Cardiomyocyte cell death; 2: Impaired myocyte contractility; 3: Left ventricle extracellular matrix remodeling (including necrosis and apoptosis); 4: Hypertrophy.

| **MOTIVE ID** | **Uniprot** | **Displayed Name** | **Protein name** |
| --- | --- | --- | --- |
| 1 | O00165 | HAX-1 | HCLS1-associated protein X-1 |
| 1 | P30405 | CyP-D | Peptidyl-prolyl cis-trans isomerase F, mitochondrial; Cyclophilin D |
| 1 | P21796 | VDAC1 | Voltage-dependent anion-selective channel protein 1 |
| 1 | P45880 | VDAC2 | Voltage-dependent anion-selective channel protein 2 |
| 1 | Q9Y277 | VDAC3 | Voltage-dependent anion-selective channel protein 3 |
| 1 | P12235 | ANT1 | ADP/ATP translocase 1 |
| 1 | P05141 | ANT2 | ADP/ATP translocase 2 |
| 1 | P12236 | ANT3 | ADP/ATP translocase 3 |
| 1 | Q9H0C2 | ANT4 | ADP/ATP translocase 4 |
| 1 | P49841 | GSK3B | Glycogen synthase kinase-3 beta |
| 1 | P25445 | FAS | Tumor necrosis factor receptor superfamily member 6 |
| 1 | P48023 | FASLG | Tumor necrosis factor ligand superfamily member 6 |
| 1 | P01375 | TNFA | Tumor necrosis factor |
| 1 | P50591 | TRAIL | Tumor necrosis factor ligand superfamily member 10 |
| 1 | Q13158 | FADD | FAS-associated death domain protein |
| 1 | Q15628 | TRADD | Tumor necrosis factor receptor type 1-associated DEATH domain protein |
| 1 | Q14790 | CASP8 | Caspase-8 |
| 1 | P42574 | CASP3 | Caspase-3 |
| 1 | P10415 | BCL2 | Apoptosis regulator Bcl-2 |
| 1 | Q07812 | Bax | Apoptosis regulator BAX |
| 1 | Q99683 | MAP3K5 / ASK-1 | Mitogen-activated protein kinase kinase kinase 5 |
| 1 | P31152 | MAPK4 | Mitogen-activated protein kinase 4 |
| 1 | Q16659 | MAPK6 | Mitogen-activated protein kinase 6 |
| 1 | P55957 | BID | BH3-interacting domain death agonist |
| 1 | P98170 | XIAP | E3 ubiquitin-protein ligase XIAP |
| 1 | Q13490 | C-IAP1 | Baculoviral IAP repeat-containing protein 2 |
| 1 | O15392 | BIRC5 | Baculoviral IAP repeat-containing protein 5 / survivin |
| 1 | Q9NR28 | DIABLO / SMAC | Diablo homolog, mitochondrial |
| 1 | O43464 | Omi/HtrA2 | Serine protease HTRA2, mitochondrial |
| 1 | Q811U4 | Mfn1 | Mitofusin-1 |
| 1 | Q07820 | MCL1 | Induced myeloid leukemia cell differentiation protein Mcl-1 |
| 1 | Q92934 | BAD | Bcl2-associated agonist of cell death |
| 1 | P04637 | p53 | Cellular tumor antigen p53 |
| 1 | P19634 | NHE1 | Sodium/hydrogen exchanger 1 |
| 1 | P29466 | CASP1 | Caspase-1 |
| 1 | P13473 | LAMP2 | Lysosome-associated membrane glycoprotein 2 |
| 1 | Q9H1Y0 | ATG5 | Autophagy protein 5 |
| 1 | O75385 | ULK1 | Serine/threonine-protein kinase ULK1 |
| 1 | P42345 | mTORC1 | Serine/threonine-protein kinase mTOR |
| 1 | P49815 | TSC2 | Tuberin |
| 1 | Q8N122 | Raptor | Regulatory-associated protein of mTOR |
| 1 | Q14457 | BECN1 | Beclin-1 |
| 1 | Q12983 | BNIP3 | BCL2/adenovirus E1B 19 kDa protein-interacting protein 3 |
| 1 | O00429 | Drp1 | Dynamin-1-like protein |
| 1 | P12644 | BMP4 | Bone morphogenetic protein 4 |
| 1 | O95140 | Mfn2 | Mitofusin-2 |
| 2 | P63098 | PPP3R1 | Calcineurin subunit B type 1 |
| 2 | Q96LZ3 | PPP3R2 | Calcineurin subunit B type 2 |
| 2 | P17612 | PKACA | cAMP-dependent protein kinase catalytic subunit alpha |
| 2 | P22694 | PKACB | cAMP-dependent protein kinase catalytic subunit beta |
| 2 | P22612 | PKACC | cAMP-dependent protein kinase catalytic subunit gamma |
| 2 | Q13237 | cGK2 | cGMP-dependent protein kinase 2 |
| 2 | Q13976 | cGK1 | cGMP-dependent protein kinase 1 |
| 2 | P78423 | CX3CL1 | Fractalkine |
| 2 | P33402 | GCSA2 | Guanylate cyclase soluble subunit alpha-2 |
| 2 | Q02108 | GCSA3 | Guanylate cyclase soluble subunit alpha-3 |
| 2 | Q02153 | GCSB1 | Guanylate cyclase soluble subunit beta-1 |
| 2 | O75343 | GCSB2 | Guanylate cyclase soluble subunit beta-2 |
| 2 | Q9UQL6 | HDAC5 | Histone deacetylase 5 |
| 2 | Q9UKV0 | HDAC9 | Histone deacetylase 9 |
| 2 | Q02078 | MEF2A | Myocyte-specific enhancer factor 2A |
| 2 | Q02080 | MEF2B | Myocyte-specific enhancer factor 2B |
| 2 | Q06413 | MEF2C | Myocyte-specific enhancer factor 2C |
| 2 | Q14814 | MEF2D | Myocyte-specific enhancer factor 2D |
| 2 | P13533 | MYH7 / aMHC | Myosin-6 (alternative name: a–myosin heavy chain) |
| 2 | P12883 | MYH6 / bMHC | Myosin-7 (alternative name: b-myosin heavy chain) |
| 2 | P29474 | NOS3 | Nitric oxide synthase, endothelial |
| 2 | O95644 | NFATc1 | Nuclear factor of activated T-cells 1 |
| 2 | Q13469 | NFATc2 | Nuclear factor of activated T-cells 2 |
| 2 | Q12968 | NFATc3 | Nuclear factor of activated T-cells 3 |
| 2 | Q14934 | NFATc4 | Nuclear factor of activated T-cells 4 |
| 2 | P26678 | PPLA | phospholamban |
| 2 | O14983 | SERCA1 | Sarcoplasmic/endoplasmic reticulum calcium ATPase 1 |
| 2 | P16615 | SERCA2 | Sarcoplasmic/endoplasmic reticulum calcium ATPase 2 |
| 2 | Q93084 | SERCA3 | Sarcoplasmic/endoplasmic reticulum calcium ATPase 3 |
| 2 | Q08209 | PPP3CA | Serine/threonine-protein phosphatase 2B catalytic subunit alpha isoform |
| 2 | P16298 | PPP3CB | Serine/threonine-protein phosphatase 2B catalytic subunit beta isoform |
| 2 | P48454 | PPP3CC | Serine/threonine-protein phosphatase 2B catalytic subunit gamma isoform |
| 2 | P10827 | THRA | Thyroid hormone receptor alpha |
| 2 | P10828 | THRB | Thyroid hormone receptor beta |
| 2 | Q8WZ42 | TTN | Titin |
| 3 | P01583 | IL1A | Interleukin-1 |
| 3 | P08254 | MMP-3 | Stromelysin-1 |
| 3 | P14780 | MMP-9 | Matrix metalloproteinase-9 |
| 3 | P03956 | MMP-1 | Interstitial collagenase |
| 3 | P16035 | TIMP2 | Metalloproteinase inhibitor 2 |
| 3 | P01137 | TGFB1 | Transforming growth factor beta-1 |
| 3 | A8TX70 | COL6A5 | Collagen alpha-5(VI) chain |
| 3 | P12109 | COL6A1 | Collagen alpha-1(VI) chain |
| 3 | P12110 | COL6A2 | Collagen alpha-2(VI) chain |
| 3 | P12111 | COL6A3 | Collagen alpha-3(VI) chain |
| 3 | P25391 | LAMA1 | Laminin subunit alpha-1 |
| 3 | P24043 | LAMA2 | Laminin subunit alpha-2 |
| 3 | Q16787 | LAMA3 | Laminin subunit alpha-3 |
| 3 | Q16363 | LAMA4 | Laminin subunit alpha-4 |
| 3 | O15230 | LAMA5 | Laminin subunit alpha-5 |
| 3 | P07942 | LAMB1 | Laminin subunit beta-1 |
| 3 | P55268 | LAMB2 | Laminin subunit beta-2 |
| 3 | Q13751 | LAMB3 | Laminin subunit beta-3 |
| 3 | A4D0S4 | LAMB4 | Laminin subunit beta-4 |
| 3 | P11047 | LAMC1 | Laminin subunit gamma-1 |
| 3 | Q13753 | LAMC2 | Laminin subunit gamma-2 |
| 3 | Q9Y6N6 | LAMC3 | Laminin subunit gamma-3 |
| 3 | P01019 | AGT | Angiotensinogen |
| 3 | P35625 | TIMP3 | Metalloproteinase inhibitor 3 |
| 3 | P16035 | TIMP4 | Metalloproteinase inhibitor 4 |
| 3 | P02751 | FN1 | Fibronectin |
| 3 | P09486 | SPARC / ON | Secreted protein acidic and rich in cysteine / Osteonectin |
| 3 | P00488 | F13A1 F13 | Transglutaminase A chain |
| 3 | P01033 | TIMP1 | Metalloproteinase inhibitor 1 |
| 3 | P09237 | MMP-7 | Matrix metalloproteinase-7 |
| 3 | P17302 | GJA1 | Gap junction alpha-1 protein / Connexin-43 |
| 3 | P12821 | ACE | Angiotensin-converting enzyme |
| 3 | P05305 | EDN1 | Endothelin-1 |
| 3 | P24821 | TN-C | Tenascin-C |
| 3 | P10451 | OPN | Osteopontin |
| 3 | P08253 | MMP-2 | Matrix metalloproteinase-2 |
| 3 | P50281 | MMP-14 | Matrix metalloproteinase-14 |
| 4 | Q9UBK2 | PPARGC1A // PGC1 | Peroxisome proliferator-activated receptor gamma coactivator 1-alpha |
| 4 | P06396 | GSN | Gelsolin |
| 4 | P49238 | CX3CR1 | CX3C chemokine receptor 1 |
| 4 | P78423 | CX3CL1 | Fractalkine |
| 4 | P49116 | NR2C2 TAK1 | Nuclear receptor subfamily 2 group C member 2 |
| 4 | P12644 | BMP4 | Bone morphogenetic protein 4 |
| 4 | P63098 | PPP3R1 | Calcineurin subunit B type 1 |
| 4 | Q96LZ3 | PPP3R2 | Calcineurin subunit B type 2 |
| 4 | P00533 | EGFR | epidermal growth factor receptor |
| 4 | P09038 | FGF-2 / bFGF | Fibroblast growth factor 2 / Basic fibroblast growth factor |
| 4 | Q14571 | IP3R2 | Inositol 1,4,5-trisphosphate receptor type 2 |
| 4 | P03956 | MMP1 | Matrix metalloproteinase-1 |
| 4 | P08235 | MR | Mineralocorticoid receptor |
| 4 | P28482 | ERK2 | Mitogen-activated protein kinase 1 |
| 4 | P27361 | ERK1 | Mitogen-activated protein kinase 3 |
| 4 | Q99836 | MyD88 | Myeloid differentiation primary response protein MyD88 |
| 4 | P29474 | NOS3 | Nitric oxide synthase, endothelial |
| 4 | O95644 | NFATc1 | Nuclear factor of activated T-cells 1 |
| 4 | Q13469 | NFATc2 | Nuclear factor of activated T-cells 2 |
| 4 | Q12968 | NFATc3 | Nuclear factor of activated T-cells 3 |
| 4 | Q14934 | NFATc4 | Nuclear factor of activated T-cells 4 |
| 4 | P61296 | HAND2 | Heart- and neural crest derivatives-expressed protein 2 |
| 4 | P05121 | PAI | Plasminogen activator inhibitor 1 |
| 4 | Q15418 | S6KA1 | Ribosomal protein S6 kinase alpha-1 |
| 4 | Q15349 | S6KA2 | Ribosomal protein S6 kinase alpha-2 |
| 4 | P51812 | S6KA3 | Ribosomal protein S6 kinase alpha-3 |
| 4 | Q08209 | PPP3CA | Serine/threonine-protein phosphatase 2B catalytic subunit alpha isoform |
| 4 | P16298 | PPP3CB | Serine/threonine-protein phosphatase 2B catalytic subunit beta isoform |
| 4 | P48454 | PPP3CC | Serine/threonine-protein phosphatase 2B catalytic subunit gamma isoform |
| 4 | Q9UBN4 | TRPC4 | Short transient receptor potential channel 4 |
| 4 | P32418 | NCX | Sodium/calcium exchanger 1 |
| 4 | P19634 | NHE1 | sodium-hydrogen antiporter1 |
| 4 | P30556 | AT1 | Type-1 angiotensin II receptor |

**Supplementary Table 2. Manual curation of the literature.** List of articles used for molecular characterization of the pathologies under study, and Sacubitril/Valsartan.

| **Manual curation of the literature:** | PMID: 26564789 | PMID: 24080184 | PMID: 19797822 |
| --- | --- | --- | --- |
|  | PMID: 26562414 | PMID: 24072174 | PMID: 19679836 |
|  | PMID: 26553996 | PMID: 23909633 | PMID: 19593942 |
|  | PMID: 26356605 | PMID: 23806284 | PMID: 19246681 |
|  | PMID: 25977452 | PMID: 23704873 | PMID: 19179805 |
|  | PMID: 25752645 | PMID: 23506867 | PMID: 19144865 |
|  | PMID: 25663976 | PMID: 23429007 | PMID: 18981739 |
|  | PMID: 25595790 | PMID: 23316962 | PMID: 18585734 |
|  | PMID: 25520329 | PMID: 23316291 | PMID: 18535174 |
|  | PMID: 25047165 | PMID: 23176689 | PMID: 18276618 |
|  | PMID: 24905188 | PMID: 22803959 | PMID: 17928585 |
|  | PMID: 24859226 | PMID: 22535253 | PMID: 17657164 |
|  | PMID: 24827991 | PMID: 21799152 | PMID: 17490677 |
|  | PMID: 24736806 | PMID: 21784127 | PMID: 17379774 |
|  | PMID: 24615014 | PMID: 21671800 | PMID: 16847152 |
|  | PMID: 24530674 | PMID: 20888832 | PMID: 15526239 |
|  | PMID: 24505034 | PMID: 20564207 | PMID: 15364610 |
|  | PMID: 24415751 | PMID: 20125030 | PMID: 14998631 |
|  | PMID: 24161931 | PMID: 19919994 | PMID: 14962485 |
|  | PMID: 24126173 | PMID: 19893013 | PMID: 11120693 |

**Supplementary Table 3: Data sources for generation and training of the mathematical models.** List of the different types of information used to generate and train the mathematical models, as well as their quantity.

| **DATA SOURCES** | **# ENTRIES** |
| --- | --- |
| Considered Interactions | 312445 |
| Considered Proteins | 15555 |
| Characterized Drugs | 6605 |
| Drug Targets | 11265 |
| Characterized Clinical Conditions | 222 |
| Clinical Conditions Key Proteins Characterized | 3712 |

**Supplementary Table 4: Molecular restrictions used for generation and training of the mathematical models.** Type and quantity of restrictions used to generate the mathematical models. ADR= Adverse Drug Reactions.

| **MAIN MODEL CONSTRUCTION RESTRICTIONS** | **DIRECT RESTRICTIONS** | |
| --- | --- | --- |
| CLINICAL CONDITION PREDICTIVE MODELS - ANNs |  | |
| Curated drug indication/ADRs database | 2500830 | |
| SAMPLING METHODS |  | |
| Curated drug indication/ADRs database | 2500830 | |
| Drug indication/ADRs protein correlations | 3970 | |
| SPECIFIC POPULATION RESTRICTIONS (High Throughput Differential Expression Restrictions) | Myocardial infarction | Heart failure |
|  | 4738 | 6074 |

| **Uniprot** | **Displayed Name** | **Protein name** | **Remodeling effector** |
| --- | --- | --- | --- |
| P31749 | AKT1 | RAC-alpha serine/threonine-protein kinase | Cardiomyocyte cell death, Impaired myocyte contractility |
| Q9Y243 | AKT3 | RAC-gamma serine/threonine-protein kinase | Cardiomyocyte cell death, Impaired myocyte contractility |
| Q05397 | FAK1 | Focal adhesion kinase 1 | Cardiomyocyte cell death, Hypertrophy, |
| P49841 | GSK3B | Glycogen synthase kinase-3 beta | Cardiomyocyte cell death |
| P27986 | P85A | Phosphatidylinositol 3-kinase regulatory subunit alpha | Cardiomyocyte cell death, Impaired myocyte contractility |
| P42336 | PK3CA | Phosphatidylinositol 4,5-bisphosphate 3-kinase catalytic subunit alpha isoform | Cardiomyocyte cell death, Impaired myocyte contractility |
| O00329 | PK3CD | Phosphatidylinositol 4,5-bisphosphate 3-kinase catalytic subunit delta isoform | Cardiomyocyte cell death, Impaired myocyte contractility |
| P10827 | THRA | Thyroid hormone receptor alpha | Impaired myocyte contractility |
| Q13490 | BIRC2 | Baculoviral IAP repeat-containing protein 2 | Cardiomyocyte cell death |
| P09038 | FGF2 | Fibroblast growth factor 2 | Hypertrophy |
| P02751 | FINC | Fibronectin | Left ventricle extracellular matrix remodeling |
| P14780 | MMP9 | Matrix metalloproteinase-9 | Left ventricle extracellular matrix remodeling |
| P42345 | MTOR | Serine/threonine-protein kinase mTOR | Cardiomyocyte cell death |
| P01137 | TGFB1 | Transforming growth factor beta-1 | Left ventricle extracellular matrix remodeling |

**Supplementary Table 5:** **Potential synergistic nodes involved in the downstream effects of sacubitril/valsartan.** The first eight proteins of the list scored the best likelihood of occupying synergistic nodes, and so are included in the MoA representation (Fig 2). However, the models predicted 6 additional proteins that could potentially act synergistically, so are included in this table as the six last proteins on the list.

**Supplementary Table 6. Specific proteins identified as only associated with the model simulating the efficacy of LCZ696 on MI patients**.

| **Uniprot** | **Displayed Name** |
| --- | --- |
| P49238 | CX3C1 (Fractalkine) |
| Q9BXN2 | CLEC7A (Dectin-1) |
| P13533 | MYH6 |
| P12883 | MYH7 |

**Supplementary Table 7: Results from pathway and GO enrichment analysis.** Sorted by p-value.

| **pathway name** | **set size** | **candidates contained (number)** | **candidates contained (%)** | **p-value** | **q-value** | **pathway source** |
| --- | --- | --- | --- | --- | --- | --- |
| G alpha (q) signalling events | 190 | 94 | 49,50% | 2,17E-145 | 1,72E-142 | Reactome |
| Gastrin-CREB signalling pathway via PKC and MAPK | 382 | 98 | 25,70% | 1,67E-118 | 6,60E-116 | Reactome |
| GPCR downstream signaling | 982 | 109 | 11,10% | 2,81E-93 | 7,43E-91 | Reactome |
| Signaling by GPCR | 1243 | 113 | 9,10% | 1,67E-88 | 3,31E-86 | Reactome |
| Class A/1 (Rhodopsin-like receptors) | 326 | 75 | 23,00% | 9,09E-82 | 1,44E-79 | Reactome |
| GPCR ligand binding | 454 | 81 | 17,90% | 8,50E-80 | 1,12E-77 | Reactome |
| Signal Transduction | 2334 | 117 | 5,00% | 3,43E-63 | 3,88E-61 | Reactome |
| Peptide ligand-binding receptors | 199 | 54 | 27,10% | 5,37E-61 | 5,32E-59 | Reactome |
| GPCRs, Class A Rhodopsin-like | 261 | 35 | 13,50% | 1,95E-27 | 1,72E-25 | Wikipathways |
| Neuroactive ligand-receptor interaction - Homo sapiens (human) | 275 | 35 | 12,70% | 1,40E-26 | 1,11E-24 | KEGG |
| Metabolism of Angiotensinogen to Angiotensins | 16 | 12 | 75,00% | 9,72E-21 | 6,44E-19 | Reactome |
| Plasma membrane estrogen receptor signaling | 42 | 16 | 38,10% | 9,76E-21 | 6,44E-19 | PID |
| Renin-angiotensin system - Homo sapiens (human) | 23 | 13 | 56,50% | 6,30E-20 | 3,84E-18 | KEGG |
| PAR1-mediated thrombin signaling events | 44 | 14 | 32,60% | 3,87E-17 | 2,19E-15 | PID |
| PAR4-mediated thrombin signaling events | 15 | 10 | 66,70% | 1,29E-16 | 6,82E-15 | PID |
| Peptide hormone metabolism | 48 | 14 | 29,20% | 2,26E-16 | 1,12E-14 | Reactome |
| Fatty Acids bound to GPR40 (FFAR1) regulate insulin secretion | 8 | 8 | 100,00% | 3,56E-16 | 1,49E-14 | Reactome |
| Free fatty acids regulate insulin secretion | 8 | 8 | 100,00% | 3,56E-16 | 1,49E-14 | Reactome |
| Orexin and neuropeptides FF and QRFP bind to their respective receptors | 8 | 8 | 100,00% | 3,56E-16 | 1,49E-14 | Reactome |
| GnRH signaling pathway - Homo sapiens (human) | 92 | 17 | 18,50% | 5,37E-16 | 2,13E-14 | KEGG |
| Platelet activation - Homo sapiens (human) | 131 | 19 | 14,50% | 1,03E-15 | 3,87E-14 | KEGG |
| Pathways in cancer - Homo sapiens (human) | 398 | 29 | 7,30% | 2,58E-15 | 9,29E-14 | KEGG |
| Platelet activation, signaling and aggregation | 229 | 23 | 10,10% | 2,73E-15 | 9,41E-14 | Reactome |
| ACE Inhibitor Pathway, Pharmacodynamics | 20 | 10 | 50,00% | 7,55E-15 | 2,39E-13 | PharmGKB |
| Agents Acting on the Renin-Angiotensin System Pathway, Pharmacodynamics | 20 | 10 | 50,00% | 7,55E-15 | 2,39E-13 | PharmGKB |
| Estrogen signaling pathway - Homo sapiens (human) | 100 | 16 | 16,00% | 4,41E-14 | 1,34E-12 | KEGG |
| Vascular smooth muscle contraction - Homo sapiens (human) | 121 | 17 | 14,00% | 6,30E-14 | 1,85E-12 | KEGG |
| Regulation of insulin secretion | 70 | 14 | 20,00% | 7,26E-14 | 2,05E-12 | Reactome |
| ACE Inhibitor Pathway | 17 | 9 | 52,90% | 9,04E-14 | 2,47E-12 | Wikipathways |
| Integration of energy metabolism | 91 | 15 | 16,50% | 1,82E-13 | 4,79E-12 | Reactome |
| Long-term depression - Homo sapiens (human) | 60 | 13 | 21,70% | 1,95E-13 | 4,99E-12 | KEGG |
| Cholinergic synapse - Homo sapiens (human) | 113 | 16 | 14,30% | 2,76E-13 | 6,83E-12 | KEGG |
| Oxytocin signaling pathway - Homo sapiens (human) | 159 | 18 | 11,30% | 5,04E-13 | 1,21E-11 | KEGG |
| Renin secretion - Homo sapiens (human) | 65 | 13 | 20,00% | 5,90E-13 | 1,38E-11 | KEGG |
| Inflammatory mediator regulation of TRP channels - Homo sapiens (human) | 99 | 15 | 15,20% | 6,58E-13 | 1,49E-11 | KEGG |
| Rap1 signaling pathway - Homo sapiens (human) | 211 | 20 | 9,50% | 6,87E-13 | 1,51E-11 | KEGG |
| Chemokine signaling pathway - Homo sapiens (human) | 189 | 19 | 10,10% | 8,57E-13 | 1,83E-11 | KEGG |
| Nongenotropic Androgen signaling | 31 | 10 | 32,30% | 1,62E-12 | 3,38E-11 | PID |
| Thromboxane A2 receptor signaling | 57 | 12 | 21,40% | 1,99E-12 | 4,03E-11 | PID |
| Gap junction - Homo sapiens (human) | 89 | 14 | 15,70% | 2,36E-12 | 4,68E-11 | KEGG |
| Acetylcholine regulates insulin secretion | 10 | 7 | 70,00% | 3,64E-12 | 7,03E-11 | Reactome |
| LPA receptor mediated events | 64 | 12 | 19,00% | 8,84E-12 | 1,67E-10 | PID |
| Endothelins | 64 | 12 | 18,80% | 1,08E-11 | 1,98E-10 | PID |
| Hemostasis | 493 | 27 | 5,50% | 2,31E-11 | 4,14E-10 | Reactome |
| P2Y receptors | 12 | 7 | 58,30% | 2,35E-11 | 4,14E-10 | Reactome |
| beta-catenin independent WNT signaling | 90 | 13 | 14,40% | 4,58E-11 | 7,89E-10 | Reactome |
| G alpha (12/13) signalling events | 76 | 12 | 16,00% | 7,66E-11 | 1,29E-09 | Reactome |
| GPCR signaling-G alpha q | 274 | 20 | 7,30% | 8,51E-11 | 1,40E-09 | INOH |
| Thrombin signalling through proteinase activated receptors (PARs) | 32 | 9 | 28,10% | 8,96E-11 | 1,45E-09 | Reactome |
| Sphingolipid signaling pathway - Homo sapiens (human) | 120 | 14 | 11,70% | 1,51E-10 | 2,40E-09 | KEGG |
| Chagas disease (American trypanosomiasis) - Homo sapiens (human) | 104 | 13 | 12,50% | 2,96E-10 | 4,60E-09 | KEGG |
| Long-term potentiation - Homo sapiens (human) | 67 | 11 | 16,40% | 3,66E-10 | 5,58E-09 | KEGG |
| S1P2 pathway | 26 | 8 | 30,80% | 4,64E-10 | 6,94E-09 | PID |
| Amoebiasis - Homo sapiens (human) | 109 | 13 | 11,90% | 5,38E-10 | 7,88E-09 | KEGG |
| Nucleotide-like (purinergic) receptors | 17 | 7 | 41,20% | 5,50E-10 | 7,92E-09 | Reactome |
| Serotonergic synapse - Homo sapiens (human) | 114 | 13 | 11,50% | 8,48E-10 | 1,20E-08 | KEGG |
| G Protein Signaling Pathways | 92 | 12 | 13,00% | 8,95E-10 | 1,24E-08 | Wikipathways |
| Nucleotide GPCRs | 11 | 6 | 54,50% | 1,20E-09 | 1,63E-08 | Wikipathways |
| Pancreatic secretion - Homo sapiens (human) | 96 | 12 | 12,50% | 1,48E-09 | 1,99E-08 | KEGG |
| Class I PI3K signaling events | 45 | 9 | 20,00% | 2,48E-09 | 3,27E-08 | PID |
| Melanogenesis - Homo sapiens (human) | 101 | 12 | 11,90% | 2,68E-09 | 3,48E-08 | KEGG |
| Calcium signaling pathway - Homo sapiens (human) | 180 | 15 | 8,30% | 3,81E-09 | 4,87E-08 | KEGG |
| IL8- and CXCR2-mediated signaling events | 34 | 8 | 23,50% | 4,98E-09 | 6,26E-08 | PID |
| HGF | 35 | 8 | 22,90% | 6,39E-09 | 7,90E-08 | INOH |
| DNA Damage Response (only ATM dependent) | 110 | 12 | 10,90% | 7,22E-09 | 8,80E-08 | Wikipathways |
| G-protein beta:gamma signalling | 51 | 9 | 17,60% | 8,01E-09 | 9,61E-08 | Reactome |
| cGMP-PKG signaling pathway - Homo sapiens (human) | 167 | 14 | 8,40% | 1,21E-08 | 1,43E-07 | KEGG |
| S1P3 pathway | 25 | 7 | 28,00% | 1,25E-08 | 1,46E-07 | PID |
| Peptide GPCRs | 73 | 10 | 13,70% | 1,48E-08 | 1,69E-07 | Wikipathways |
| EPHB forward signaling | 39 | 8 | 20,50% | 1,60E-08 | 1,69E-07 | PID |
| Proteoglycans in cancer - Homo sapiens (human) | 204 | 15 | 7,50% | 1,72E-08 | 1,69E-07 | KEGG |
| Angiotensin Metabolism | 4 | 4 | 100,00% | 2,01E-08 | 1,69E-07 | SMPDB |
| Benazepril Action Pathway | 4 | 4 | 100,00% | 2,01E-08 | 1,69E-07 | SMPDB |
| Candesartan Action Pathway | 4 | 4 | 100,00% | 2,01E-08 | 1,69E-07 | SMPDB |
| Captopril Action Pathway | 4 | 4 | 100,00% | 2,01E-08 | 1,69E-07 | SMPDB |
| Cilazapril Action Pathway | 4 | 4 | 100,00% | 2,01E-08 | 1,69E-07 | SMPDB |
| Enalapril Action Pathway | 4 | 4 | 100,00% | 2,01E-08 | 1,69E-07 | SMPDB |
| Eprosartan Action Pathway | 4 | 4 | 100,00% | 2,01E-08 | 1,69E-07 | SMPDB |
| Forasartan Action Pathway | 4 | 4 | 100,00% | 2,01E-08 | 1,69E-07 | SMPDB |
| Fosinopril Action Pathway | 4 | 4 | 100,00% | 2,01E-08 | 1,69E-07 | SMPDB |
| Irbesartan Action Pathway | 4 | 4 | 100,00% | 2,01E-08 | 1,69E-07 | SMPDB |
| Lisinopril Action Pathway | 4 | 4 | 100,00% | 2,01E-08 | 1,69E-07 | SMPDB |
| Losartan Action Pathway | 4 | 4 | 100,00% | 2,01E-08 | 1,69E-07 | SMPDB |
| Moexipril Action Pathway | 4 | 4 | 100,00% | 2,01E-08 | 1,69E-07 | SMPDB |
| Olmesartan Action Pathway | 4 | 4 | 100,00% | 2,01E-08 | 1,69E-07 | SMPDB |
| Perindopril Action Pathway | 4 | 4 | 100,00% | 2,01E-08 | 1,69E-07 | SMPDB |
| Quinapril Action Pathway | 4 | 4 | 100,00% | 2,01E-08 | 1,69E-07 | SMPDB |
| Ramipril Action Pathway | 4 | 4 | 100,00% | 2,01E-08 | 1,69E-07 | SMPDB |
| Rescinnamine Action Pathway | 4 | 4 | 100,00% | 2,01E-08 | 1,69E-07 | SMPDB |
| Spirapril Action Pathway | 4 | 4 | 100,00% | 2,01E-08 | 1,69E-07 | SMPDB |
| Telmisartan Action Pathway | 4 | 4 | 100,00% | 2,01E-08 | 1,69E-07 | SMPDB |
| Temocapril Action Pathway | 4 | 4 | 100,00% | 2,01E-08 | 1,69E-07 | SMPDB |
| Trandolapril Action Pathway | 4 | 4 | 100,00% | 2,01E-08 | 1,69E-07 | SMPDB |
| Valsartan Action Pathway | 4 | 4 | 100,00% | 2,01E-08 | 1,69E-07 | SMPDB |
| thrombin signaling and protease-activated receptors | 27 | 7 | 25,90% | 2,27E-08 | 1,89E-07 | BioCarta |
| Phosphatidylinositol signaling system - Homo sapiens (human) | 98 | 11 | 11,20% | 2,33E-08 | 1,92E-07 | KEGG |
| GPCR GroupI metabotropic glutamate receptor | 28 | 7 | 25,00% | 3,00E-08 | 2,41E-07 | INOH |
| IL8- and CXCR1-mediated signaling events | 28 | 7 | 25,00% | 3,00E-08 | 2,41E-07 | PID |
| Bisphosphonate Pathway, Pharmacodynamics | 17 | 6 | 35,30% | 3,02E-08 | 2,41E-07 | PharmGKB |
| G alpha (i) signalling events | 243 | 16 | 6,60% | 3,30E-08 | 2,61E-07 | Reactome |
| Glucagon signaling pathway - Homo sapiens (human) | 102 | 11 | 10,80% | 3,56E-08 | 2,79E-07 | KEGG |
| VEGF signaling pathway - Homo sapiens (human) | 61 | 9 | 14,80% | 4,13E-08 | 3,20E-07 | KEGG |
| Beta-agonist/Beta-blocker Pathway, Pharmacodynamics | 66 | 9 | 13,80% | 7,30E-08 | 5,58E-07 | PharmGKB |
| Insulin secretion - Homo sapiens (human) | 86 | 10 | 11,60% | 7,40E-08 | 5,58E-07 | KEGG |
| CXCR4-mediated signaling events | 88 | 10 | 11,60% | 7,40E-08 | 5,58E-07 | PID |
| Serotonin Receptor 2 and ELK-SRF-GATA4 signaling | 20 | 6 | 30,00% | 9,17E-08 | 6,85E-07 | Wikipathways |
| Activation of RAS in B cells | 5 | 4 | 80,00% | 9,95E-08 | 7,36E-07 | Reactome |
| Signaling by FGFR4 in disease | 11 | 5 | 45,50% | 1,02E-07 | 7,50E-07 | Reactome |
| Platelet Aggregation Inhibitor Pathway, Pharmacodynamics | 49 | 8 | 16,30% | 1,06E-07 | 7,72E-07 | PharmGKB |
| Glutamatergic synapse - Homo sapiens (human) | 116 | 11 | 9,60% | 1,24E-07 | 8,96E-07 | KEGG |
| RalA downstream regulated genes | 12 | 5 | 41,70% | 1,74E-07 | 1,24E-06 | Wikipathways |
| Thyroid hormone signaling pathway - Homo sapiens (human) | 119 | 11 | 9,20% | 1,77E-07 | 1,25E-06 | KEGG |
| Thyroid hormone synthesis - Homo sapiens (human) | 72 | 9 | 12,50% | 1,81E-07 | 1,27E-06 | KEGG |
| roles of arrestin dependent recruitment of src kinases in gpcr signaling | 36 | 7 | 19,40% | 1,95E-07 | 1,36E-06 | BioCarta |
| GPVI-mediated activation cascade | 54 | 8 | 14,80% | 2,33E-07 | 1,60E-06 | Reactome |
| Gastric acid secretion - Homo sapiens (human) | 75 | 9 | 12,00% | 2,59E-07 | 1,77E-06 | KEGG |
| Regulation of actin cytoskeleton - Homo sapiens (human) | 215 | 14 | 6,50% | 2,73E-07 | 1,85E-06 | KEGG |
| CXCR3-mediated signaling events | 38 | 7 | 18,40% | 2,89E-07 | 1,94E-06 | PID |
| Vasopressin-like receptors | 6 | 4 | 66,70% | 2,96E-07 | 1,97E-06 | Reactome |
| GPCR signaling-G alpha s PKA and ERK | 285 | 16 | 5,60% | 2,98E-07 | 1,97E-06 | INOH |
| ADP signalling through P2Y purinoceptor 1 | 24 | 6 | 25,00% | 3,06E-07 | 1,99E-06 | Reactome |
| Sema4D induced cell migration and growth-cone collapse | 24 | 6 | 25,00% | 3,06E-07 | 1,99E-06 | Reactome |
| Alzheimers Disease | 144 | 9 | 11,50% | 3,65E-07 | 2,35E-06 | Wikipathways |
| ErbB2/ErbB3 signaling events | 40 | 7 | 17,50% | 4,19E-07 | 2,67E-06 | PID |
| Constitutive Signaling by EGFRvIII | 14 | 5 | 35,70% | 4,30E-07 | 2,71E-06 | Reactome |
| Signaling by EGFRvIII in Cancer | 14 | 5 | 35,70% | 4,30E-07 | 2,71E-06 | Reactome |
| Constitutive Signaling by Ligand-Responsive EGFR Cancer Variants | 15 | 5 | 33,30% | 6,39E-07 | 3,93E-06 | Reactome |
| Signaling by EGFR in Cancer | 15 | 5 | 33,30% | 6,39E-07 | 3,93E-06 | Reactome |
| Signaling by Ligand-Responsive EGFR Variants in Cancer | 15 | 5 | 33,30% | 6,39E-07 | 3,93E-06 | Reactome |
| Sema4D in semaphorin signaling | 27 | 6 | 22,20% | 6,54E-07 | 3,98E-06 | Reactome |
| Neurotrophic factor-mediated Trk receptor signaling | 63 | 8 | 12,70% | 7,92E-07 | 4,79E-06 | PID |
| GPCR signaling-cholera toxin | 270 | 15 | 5,60% | 8,21E-07 | 4,91E-06 | INOH |
| Selective Serotonin Reuptake Inhibitor Pathway, Pharmacodynamics | 28 | 6 | 21,40% | 8,24E-07 | 4,91E-06 | PharmGKB |
| GPCR signaling-G alpha s Epac and ERK | 272 | 15 | 5,50% | 9,02E-07 | 5,33E-06 | INOH |
| PLC beta mediated events | 46 | 7 | 15,20% | 1,13E-06 | 6,59E-06 | Reactome |
| Regulation of Microtubule Cytoskeleton | 46 | 7 | 15,20% | 1,13E-06 | 6,59E-06 | Wikipathways |
| TSH signaling pathway | 66 | 8 | 12,10% | 1,14E-06 | 6,59E-06 | Wikipathways |
| Salivary secretion - Homo sapiens (human) | 90 | 9 | 10,00% | 1,25E-06 | 7,19E-06 | KEGG |
| G-protein mediated events | 47 | 7 | 14,90% | 1,32E-06 | 7,50E-06 | Reactome |
| superpathway of inositol phosphate compounds | 69 | 8 | 11,80% | 1,44E-06 | 8,13E-06 | HumanCyc |
| Signal amplification | 31 | 6 | 19,40% | 1,56E-06 | 8,78E-06 | Reactome |
| Adrenergic signaling in cardiomyocytes - Homo sapiens (human) | 149 | 11 | 7,40% | 1,71E-06 | 9,45E-06 | KEGG |
| Regulation of Actin Cytoskeleton | 151 | 11 | 7,40% | 1,71E-06 | 9,45E-06 | Wikipathways |
| EPHA2 forward signaling | 18 | 5 | 27,80% | 1,77E-06 | 9,68E-06 | PID |
| Tie2 Signaling | 18 | 5 | 27,80% | 1,77E-06 | 9,68E-06 | Reactome |
| Fc epsilon RI signaling pathway - Homo sapiens (human) | 70 | 8 | 11,40% | 1,80E-06 | 9,76E-06 | KEGG |
| Circadian entrainment - Homo sapiens (human) | 96 | 9 | 9,40% | 2,16E-06 | 1,17E-05 | KEGG |
| B cell receptor signaling pathway - Homo sapiens (human) | 72 | 8 | 11,10% | 2,24E-06 | 1,20E-05 | KEGG |
| bioactive peptide induced signaling pathway | 33 | 6 | 18,20% | 2,31E-06 | 1,22E-05 | BioCarta |
| Netrin-mediated signaling events | 33 | 6 | 18,20% | 2,31E-06 | 1,22E-05 | PID |
| Ca2+ pathway | 51 | 7 | 13,70% | 2,33E-06 | 1,22E-05 | Reactome |
| PDGFR-beta signaling pathway | 126 | 10 | 7,90% | 2,68E-06 | 1,40E-05 | PID |
| African trypanosomiasis - Homo sapiens (human) | 34 | 6 | 17,60% | 2,77E-06 | 1,43E-05 | KEGG |
| GPCR signaling-G alpha i | 262 | 14 | 5,30% | 3,09E-06 | 1,58E-05 | INOH |
| GPCR signaling-pertussis toxin | 262 | 14 | 5,30% | 3,09E-06 | 1,58E-05 | INOH |
| ras signaling pathway | 20 | 5 | 25,00% | 3,14E-06 | 1,60E-05 | BioCarta |
| angiotensin ii mediated activation of jnk pathway via pyk2 dependent signaling | 35 | 6 | 17,10% | 3,31E-06 | 1,65E-05 | BioCarta |
| Choline metabolism in cancer - Homo sapiens (human) | 101 | 9 | 8,90% | 3,31E-06 | 1,65E-05 | KEGG |
| Arf6 signaling events | 35 | 6 | 17,10% | 3,31E-06 | 1,65E-05 | PID |
| Signalling by NGF | 386 | 17 | 4,40% | 3,39E-06 | 1,68E-05 | Reactome |
| CD4 T cell receptor signaling | 130 | 10 | 7,70% | 3,56E-06 | 1,74E-05 | INOH |
| Dopaminergic synapse - Homo sapiens (human) | 131 | 10 | 7,70% | 3,56E-06 | 1,74E-05 | KEGG |
| Retrograde endocannabinoid signaling - Homo sapiens (human) | 103 | 9 | 8,80% | 3,60E-06 | 1,75E-05 | KEGG |
| Trk receptor signaling mediated by PI3K and PLC-gamma | 36 | 6 | 16,70% | 3,94E-06 | 1,90E-05 | PID |
| E-cadherin signaling in keratinocytes | 21 | 5 | 23,80% | 4,09E-06 | 1,96E-05 | PID |
| Signaling by FGFR3 in disease | 22 | 5 | 22,70% | 5,24E-06 | 2,48E-05 | Reactome |
| Signaling by FGFR3 mutants | 22 | 5 | 22,70% | 5,24E-06 | 2,48E-05 | Reactome |
| Internalization of ErbB1 | 38 | 6 | 15,80% | 5,47E-06 | 2,58E-05 | PID |
| cxcr4 signaling pathway | 11 | 4 | 36,40% | 6,21E-06 | 2,87E-05 | BioCarta |
| Hormone ligand-binding receptors | 11 | 4 | 36,40% | 6,21E-06 | 2,87E-05 | Reactome |
| p38MAPK events | 11 | 4 | 36,40% | 6,21E-06 | 2,87E-05 | Reactome |
| Signaling events regulated by Ret tyrosine kinase | 39 | 6 | 15,40% | 6,40E-06 | 2,95E-05 | PID |
| Opioid Signalling | 84 | 8 | 9,60% | 6,60E-06 | 3,00E-05 | Reactome |
| ccr3 signaling in eosinophils | 23 | 5 | 21,70% | 6,63E-06 | 3,00E-05 | BioCarta |
| Thromboxane signalling through TP receptor | 23 | 5 | 21,70% | 6,63E-06 | 3,00E-05 | Reactome |
| Wnt signaling pathway - Homo sapiens (human) | 140 | 10 | 7,10% | 6,92E-06 | 3,12E-05 | KEGG |
| CD4 T cell receptor signaling-ERK cascade | 41 | 6 | 14,60% | 8,65E-06 | 3,87E-05 | INOH |
| Colorectal cancer - Homo sapiens (human) | 62 | 7 | 11,30% | 8,86E-06 | 3,94E-05 | KEGG |
| Fc Epsilon Receptor I Signaling in Mast Cells | 42 | 6 | 14,30% | 9,99E-06 | 4,42E-05 | SMPDB |
| Effects of PIP2 hydrolysis | 25 | 5 | 20,00% | 1,03E-05 | 4,52E-05 | Reactome |
| activation of pkc through g-protein coupled receptors | 13 | 4 | 30,80% | 1,32E-05 | 5,78E-05 | BioCarta |
| Renal cell carcinoma - Homo sapiens (human) | 66 | 7 | 10,60% | 1,35E-05 | 5,80E-05 | KEGG |
| C-type lectin receptors (CLRs) | 66 | 7 | 10,60% | 1,35E-05 | 5,80E-05 | Reactome |
| Rac1-Pak1-p38-MMP-2 pathway | 66 | 7 | 10,60% | 1,35E-05 | 5,80E-05 | Wikipathways |
| corticosteroids and cardioprotection | 27 | 5 | 18,50% | 1,53E-05 | 6,45E-05 | BioCarta |
| Sorafenib Pharmacodynamics | 27 | 5 | 18,50% | 1,53E-05 | 6,45E-05 | PharmGKB |
| Signaling by FGFR2 in disease | 27 | 5 | 18,50% | 1,53E-05 | 6,45E-05 | Reactome |
| Signaling by FGFR2 mutants | 27 | 5 | 18,50% | 1,53E-05 | 6,45E-05 | Reactome |
| phospholipase c signaling pathway | 5 | 3 | 60,00% | 1,68E-05 | 6,95E-05 | BioCarta |
| Free fatty acid receptors | 5 | 3 | 60,00% | 1,68E-05 | 6,95E-05 | Reactome |
| negative regulation of TCF-dependent signaling by DVL-interacting proteins | 5 | 3 | 60,00% | 1,68E-05 | 6,95E-05 | Reactome |
| Proton Pump Inhibitor Pathway, Pharmacodynamics | 46 | 6 | 13,00% | 1,71E-05 | 7,07E-05 | PharmGKB |
| influence of ras and rho proteins on g1 to s transition | 28 | 5 | 17,90% | 1,85E-05 | 7,53E-05 | BioCarta |
| Signaling by FGFR4 mutants | 28 | 5 | 17,90% | 1,85E-05 | 7,53E-05 | Reactome |
| Regulation of RhoA activity | 48 | 6 | 12,80% | 1,95E-05 | 7,87E-05 | PID |
| PCP/CE pathway | 47 | 6 | 12,80% | 1,95E-05 | 7,87E-05 | Reactome |
| G alpha (s) signalling events | 128 | 9 | 7,10% | 2,16E-05 | 8,69E-05 | Reactome |
| Endocrine and other factor-regulated calcium reabsorption - Homo sapiens (human) | 48 | 6 | 12,50% | 2,20E-05 | 8,77E-05 | KEGG |
| G beta:gamma signalling through PI3Kgamma | 48 | 6 | 12,50% | 2,20E-05 | 8,77E-05 | Reactome |
| Prolactin signaling pathway - Homo sapiens (human) | 72 | 7 | 9,70% | 2,40E-05 | 9,51E-05 | KEGG |
| -arrestins in gpcr desensitization | 30 | 5 | 16,70% | 2,62E-05 | 0,000103 | BioCarta |
| Signaling by Wnt | 278 | 13 | 4,70% | 2,83E-05 | 0,000111 | Reactome |
| B cell receptor signaling | 102 | 8 | 7,80% | 3,03E-05 | 0,000118 | INOH |
| activation of camp-dependent protein kinase pka | 31 | 5 | 16,10% | 3,10E-05 | 0,00012 | BioCarta |
| T cell receptor signaling pathway - Homo sapiens (human) | 104 | 8 | 7,70% | 3,49E-05 | 0,000135 | KEGG |
| Endometrial cancer - Homo sapiens (human) | 52 | 6 | 11,50% | 3,51E-05 | 0,000135 | KEGG |
| role of -arrestins in the activation and targeting of map kinases | 32 | 5 | 15,60% | 3,64E-05 | 0,000138 | BioCarta |
| Noncanonical Wnt signaling pathway | 32 | 5 | 15,60% | 3,64E-05 | 0,000138 | PID |
| Osteopontin-mediated events | 32 | 5 | 15,60% | 3,64E-05 | 0,000138 | PID |
| ErbB1 downstream signaling | 107 | 8 | 7,50% | 4,00E-05 | 0,000151 | PID |
| Bacterial invasion of epithelial cells - Homo sapiens (human) | 78 | 7 | 9,00% | 4,06E-05 | 0,000152 | KEGG |
| aspirin blocks signaling pathway involved in platelet activation | 17 | 4 | 23,50% | 4,23E-05 | 0,000156 | BioCarta |
| role of pi3k subunit p85 in regulation of actin organization and cell migration | 17 | 4 | 23,50% | 4,23E-05 | 0,000156 | BioCarta |
| phospholipids as signalling intermediaries | 33 | 5 | 15,20% | 4,25E-05 | 0,000156 | BioCarta |
| role of egf receptor transactivation by gpcrs in cardiac hypertrophy | 33 | 5 | 15,20% | 4,25E-05 | 0,000156 | BioCarta |
| Alpha 6 Beta 4 signaling pathway | 33 | 5 | 15,20% | 4,25E-05 | 0,000156 | Wikipathways |
| IL2-mediated signaling events | 54 | 6 | 11,10% | 4,37E-05 | 0,00016 | PID |
| Signaling events mediated by Hepatocyte Growth Factor Receptor (c-Met) | 82 | 7 | 8,60% | 5,18E-05 | 0,000188 | PID |
| Signaling pathways regulating pluripotency of stem cells - Homo sapiens (human) | 142 | 9 | 6,30% | 5,23E-05 | 0,000189 | KEGG |
| Non-small cell lung cancer - Homo sapiens (human) | 56 | 6 | 10,70% | 5,39E-05 | 0,000194 | KEGG |
| p75 NTR receptor-mediated signalling | 83 | 7 | 8,50% | 5,61E-05 | 0,000201 | Reactome |
| Acute myeloid leukemia - Homo sapiens (human) | 57 | 6 | 10,50% | 5,96E-05 | 0,000213 | KEGG |
| MicroRNAs in cardiomyocyte hypertrophy | 102 | 7 | 8,40% | 6,07E-05 | 0,000216 | Wikipathways |
| rac1 cell motility signaling pathway | 37 | 5 | 13,90% | 6,55E-05 | 0,000228 | BioCarta |
| IGF signaling | 36 | 5 | 13,90% | 6,55E-05 | 0,000228 | INOH |
| E-cadherin signaling in the nascent adherens junction | 36 | 5 | 13,90% | 6,55E-05 | 0,000228 | PID |
| Signaling by FGFR1 in disease | 36 | 5 | 13,90% | 6,55E-05 | 0,000228 | Reactome |
| Signaling by FGFR1 mutants | 36 | 5 | 13,90% | 6,55E-05 | 0,000228 | Reactome |
| SHP2 signaling | 58 | 6 | 10,30% | 6,59E-05 | 0,000228 | PID |
| D-myo-inositol-5-phosphate metabolism | 19 | 4 | 21,10% | 6,77E-05 | 0,000233 | HumanCyc |
| GMCSF-mediated signaling events | 37 | 5 | 13,50% | 7,50E-05 | 0,000257 | PID |
| ErbB signaling pathway - Homo sapiens (human) | 87 | 7 | 8,00% | 8,21E-05 | 0,00028 | KEGG |
| VEGFA-VEGFR2 Pathway | 266 | 12 | 4,50% | 8,23E-05 | 0,00028 | Reactome |
| G beta:gamma signalling through PLC beta | 20 | 4 | 20,00% | 8,38E-05 | 0,000284 | Reactome |
| Ras signaling pathway - Homo sapiens (human) | 228 | 11 | 4,80% | 9,03E-05 | 0,000304 | KEGG |
| MAP kinase cascade | 8 | 3 | 37,50% | 9,14E-05 | 0,000307 | HumanCyc |
| Signaling events mediated by focal adhesion kinase | 62 | 6 | 9,70% | 9,62E-05 | 0,000321 | PID |
| Neurotrophin signaling pathway - Homo sapiens (human) | 120 | 8 | 6,70% | 9,69E-05 | 0,000322 | KEGG |
| Ghrelin | 39 | 5 | 12,80% | 9,72E-05 | 0,000322 | NetPath |
| pkc-catalyzed phosphorylation of inhibitory phosphoprotein of myosin phosphatase | 22 | 4 | 19,00% | 0,000103 | 0,000336 | BioCarta |
| Sphingosine 1-phosphate (S1P) pathway | 21 | 4 | 19,00% | 0,000103 | 0,000336 | PID |
| Presynaptic function of Kainate receptors | 21 | 4 | 19,00% | 0,000103 | 0,000336 | Reactome |
| Signaling by VEGF | 274 | 12 | 4,40% | 0,000109 | 0,000356 | Reactome |
| Rho GTPase cycle | 125 | 8 | 6,50% | 0,000115 | 0,000374 | Reactome |
| cystic fibrosis transmembrane conductance regulator (cftr) and beta 2 adrenergic receptor (b2ar) pathway | 22 | 4 | 18,20% | 0,000124 | 0,0004 | BioCarta |
| Inositol phosphate metabolism | 41 | 5 | 12,20% | 0,000124 | 0,0004 | INOH |
| Glioma - Homo sapiens (human) | 65 | 6 | 9,20% | 0,000125 | 0,000402 | KEGG |
| Elevation of cytosolic Ca2+ levels | 9 | 3 | 33,30% | 0,000136 | 0,000432 | Reactome |
| SHC-related events triggered by IGF1R | 9 | 3 | 33,30% | 0,000136 | 0,000432 | Reactome |
| Pancreatic cancer - Homo sapiens (human) | 66 | 6 | 9,10% | 0,000137 | 0,000433 | KEGG |
| chrebp regulation by carbohydrates and camp | 42 | 5 | 11,90% | 0,00014 | 0,000439 | BioCarta |
| EPHB-mediated forward signaling | 42 | 5 | 11,90% | 0,00014 | 0,000439 | Reactome |
| cAMP signaling pathway - Homo sapiens (human) | 200 | 10 | 5,00% | 0,000147 | 0,000461 | KEGG |
| Central carbon metabolism in cancer - Homo sapiens (human) | 67 | 6 | 9,00% | 0,000149 | 0,000461 | KEGG |
| Signaling events mediated by PRL | 23 | 4 | 17,40% | 0,000149 | 0,000461 | PID |
| Semaphorin interactions | 67 | 6 | 9,00% | 0,000149 | 0,000461 | Reactome |
| activation of csk by camp-dependent protein kinase inhibits signaling through the t cell receptor | 45 | 5 | 11,60% | 0,000156 | 0,000482 | BioCarta |
| Signaling by the B Cell Receptor (BCR) | 170 | 9 | 5,40% | 0,000174 | 0,000534 | Reactome |
| PDGF | 44 | 5 | 11,40% | 0,000175 | 0,000534 | INOH |
| Ephrin B reverse signaling | 24 | 4 | 16,70% | 0,000177 | 0,000539 | PID |
| Alzheimer,s disease - Homo sapiens (human) | 168 | 9 | 5,40% | 0,00019 | 0,000578 | KEGG |
| EGFR Transactivation by Gastrin | 10 | 3 | 30,00% | 0,000192 | 0,00058 | Reactome |
| WNT mediated activation of DVL | 10 | 3 | 30,00% | 0,000192 | 0,00058 | Reactome |
| NRAGE signals death through JNK | 45 | 5 | 11,10% | 0,000195 | 0,000582 | Reactome |
| Signaling by FGFR in disease | 45 | 5 | 11,10% | 0,000195 | 0,000582 | Reactome |
| Cell surface interactions at the vascular wall | 101 | 7 | 7,00% | 0,000198 | 0,000588 | Reactome |
| Melanoma - Homo sapiens (human) | 71 | 6 | 8,50% | 0,000205 | 0,000606 | KEGG |
| CDC42 signaling events | 71 | 6 | 8,50% | 0,000205 | 0,000606 | PID |
| D-myo-inositol (1,4,5)-trisphosphate biosynthesis | 26 | 4 | 16,00% | 0,000209 | 0,00061 | HumanCyc |
| Natural killer cell mediated cytotoxicity - Homo sapiens (human) | 134 | 8 | 6,00% | 0,000209 | 0,00061 | KEGG |
| RAF activation | 25 | 4 | 16,00% | 0,000209 | 0,00061 | Reactome |
| Role of phospholipids in phagocytosis | 71 | 5 | 10,90% | 0,000216 | 0,00063 | Reactome |
| Chronic myeloid leukemia - Homo sapiens (human) | 73 | 6 | 8,20% | 0,000239 | 0,000693 | KEGG |
| ion channels and their functional role in vascular endothelium | 47 | 5 | 10,60% | 0,00024 | 0,000693 | BioCarta |
| links between pyk2 and map kinases | 26 | 4 | 15,40% | 0,000244 | 0,000701 | BioCarta |
| TSH | 26 | 4 | 15,40% | 0,000244 | 0,000701 | NetPath |
| insulin signaling pathway | 11 | 3 | 27,30% | 0,000262 | 0,000747 | BioCarta |
| RHO GTPases activate KTN1 | 11 | 3 | 27,30% | 0,000262 | 0,000747 | Reactome |
| transcription factor creb and its extracellular signals | 27 | 4 | 14,80% | 0,000284 | 0,000801 | BioCarta |
| 3-phosphoinositide biosynthesis | 28 | 4 | 14,80% | 0,000284 | 0,000801 | HumanCyc |
| Synthesis of IP3 and IP4 in the cytosol | 27 | 4 | 14,80% | 0,000284 | 0,000801 | Reactome |
| Angiopoietin receptor Tie2-mediated signaling | 50 | 5 | 10,00% | 0,000322 | 0,000904 | PID |
| g-protein signaling through tubby proteins | 12 | 3 | 25,00% | 0,000347 | 0,000967 | BioCarta |
| degradation of DVL | 12 | 3 | 25,00% | 0,000347 | 0,000967 | Reactome |
| Wnt Signaling Pathway Netpath | 51 | 5 | 9,80% | 0,000353 | 0,000982 | Wikipathways |
| Nephrin/Neph1 signaling in the kidney podocyte | 29 | 4 | 13,80% | 0,000378 | 0,00104 | PID |
| MAPK Cascade | 29 | 4 | 13,80% | 0,000378 | 0,00104 | Wikipathways |
| Calcium Regulation in the Cardiac Cell | 149 | 8 | 5,40% | 0,000429 | 0,00117 | Wikipathways |
| fmlp induced chemokine gene expression in hmc-1 cells | 30 | 4 | 13,30% | 0,000432 | 0,00117 | BioCarta |
| vegf hypoxia and angiogenesis | 30 | 4 | 13,30% | 0,000432 | 0,00117 | BioCarta |
| Nectin adhesion pathway | 30 | 4 | 13,30% | 0,000432 | 0,00117 | PID |
| Glucagon-type ligand receptors | 30 | 4 | 13,30% | 0,000432 | 0,00117 | Reactome |
| Extracellular vesicle-mediated signaling in recipient cells | 30 | 4 | 13,30% | 0,000432 | 0,00117 | Wikipathways |
| multiple antiapoptotic pathways from igf-1r signaling lead to bad phosphorylation | 13 | 3 | 23,10% | 0,000447 | 0,0012 | BioCarta |
| trka receptor signaling pathway | 13 | 3 | 23,10% | 0,000447 | 0,0012 | BioCarta |
| CLEC7A (Dectin-1) induces NFAT activation | 13 | 3 | 23,10% | 0,000447 | 0,0012 | Reactome |
| Signaling Pathways in Glioblastoma | 83 | 6 | 7,30% | 0,00045 | 0,0012 | Wikipathways |
| CD4 T cell receptor signaling-JNK cascade | 54 | 5 | 9,30% | 0,000462 | 0,00123 | INOH |
| erk and pi-3 kinase are necessary for collagen binding in corneal epithelia | 31 | 4 | 12,90% | 0,000491 | 0,0013 | BioCarta |
| Activation of Kainate Receptors upon glutamate binding | 32 | 4 | 12,50% | 0,000556 | 0,00146 | Reactome |
| CD28 co-stimulation | 32 | 4 | 12,50% | 0,000556 | 0,00146 | Reactome |
| Myometrial Relaxation and Contraction Pathways | 155 | 8 | 5,20% | 0,000558 | 0,00146 | Wikipathways |
| Atypical NF-kappaB pathway | 14 | 3 | 21,40% | 0,000564 | 0,00147 | PID |
| Ras signaling in the CD4+ TCR pathway | 14 | 3 | 21,40% | 0,000564 | 0,00147 | PID |
| DAP12 signaling | 282 | 11 | 3,90% | 0,000572 | 0,00149 | Reactome |
| Regulation of Ras family activation | 34 | 4 | 12,10% | 0,000627 | 0,00161 | PID |
| Glucagon signaling in metabolic regulation | 33 | 4 | 12,10% | 0,000627 | 0,00161 | Reactome |
| Synthesis of PIPs at the plasma membrane | 33 | 4 | 12,10% | 0,000627 | 0,00161 | Reactome |
| Serotonin HTR1 Group and FOS Pathway | 33 | 4 | 12,10% | 0,000627 | 0,00161 | Wikipathways |
| Prostate cancer - Homo sapiens (human) | 89 | 6 | 6,70% | 0,000698 | 0,00177 | KEGG |
| Arf6 downstream pathway | 15 | 3 | 20,00% | 0,000698 | 0,00177 | PID |
| DCC mediated attractive signaling | 15 | 3 | 20,00% | 0,000698 | 0,00177 | Reactome |
| GPCRs, Other | 100 | 6 | 6,60% | 0,000785 | 0,00199 | Wikipathways |
| Cell death signalling via NRAGE, NRIF and NADE | 61 | 5 | 8,20% | 0,000813 | 0,00205 | Reactome |
| Axon guidance - Homo sapiens (human) | 127 | 7 | 5,50% | 0,000844 | 0,00212 | KEGG |
| Viral carcinogenesis - Homo sapiens (human) | 206 | 9 | 4,40% | 0,000847 | 0,00212 | KEGG |
| control of skeletal myogenesis by hdac and calcium/calmodulin-dependent kinase (camk) | 16 | 3 | 18,80% | 0,000852 | 0,00212 | BioCarta |
| the igf-1 receptor and longevity | 17 | 3 | 18,80% | 0,000852 | 0,00212 | BioCarta |
| N-cadherin signaling events | 36 | 4 | 11,10% | 0,000878 | 0,00218 | PID |
| DAP12 interactions | 298 | 11 | 3,70% | 0,000904 | 0,00224 | Reactome |
| Integrins in angiogenesis | 63 | 5 | 7,90% | 0,000942 | 0,00233 | PID |
| trefoil factors initiate mucosal healing | 37 | 4 | 10,80% | 0,000975 | 0,0024 | BioCarta |
| Fcgamma receptor (FCGR) dependent phagocytosis | 120 | 6 | 6,30% | 0,000984 | 0,00241 | Reactome |
| role of nicotinic acetylcholine receptors in the regulation of apoptosis | 17 | 3 | 17,60% | 0,00103 | 0,0025 | BioCarta |
| ucalpain and friends in cell spread | 17 | 3 | 17,60% | 0,00103 | 0,0025 | BioCarta |
| Regulation of RAC1 activity | 39 | 4 | 10,50% | 0,00108 | 0,00261 | PID |
| MAP2K and MAPK activation | 38 | 4 | 10,50% | 0,00108 | 0,00261 | Reactome |
| G13 Signaling Pathway | 38 | 4 | 10,50% | 0,00108 | 0,00261 | Wikipathways |
| Fc epsilon receptor (FCERI) signaling | 330 | 11 | 3,60% | 0,00109 | 0,00263 | Reactome |
| Axon guidance | 459 | 14 | 3,10% | 0,00116 | 0,00279 | Reactome |
| Wnt Signaling Pathway | 66 | 5 | 7,60% | 0,00116 | 0,00279 | Wikipathways |
| phospholipases | 39 | 4 | 10,30% | 0,00119 | 0,00283 | HumanCyc |
| Aldosterone-regulated sodium reabsorption - Homo sapiens (human) | 39 | 4 | 10,30% | 0,00119 | 0,00283 | KEGG |
| nerve growth factor pathway (ngf) | 18 | 3 | 16,70% | 0,00122 | 0,00288 | BioCarta |
| Pathway_PA165959425 | 18 | 3 | 16,70% | 0,00122 | 0,00288 | PharmGKB |
| SHC-mediated cascade:FGFR3 | 18 | 3 | 16,70% | 0,00122 | 0,00288 | Reactome |
| FGF | 67 | 5 | 7,50% | 0,00125 | 0,00291 | INOH |
| EGFR Inhibitor Pathway, Pharmacodynamics | 67 | 5 | 7,50% | 0,00125 | 0,00291 | PharmGKB |
| NGF signalling via TRKA from the plasma membrane | 310 | 11 | 3,60% | 0,00125 | 0,00291 | Reactome |
| Downstream signaling of activated FGFR1 | 267 | 10 | 3,80% | 0,00139 | 0,0032 | Reactome |
| Downstream signaling of activated FGFR2 | 267 | 10 | 3,80% | 0,00139 | 0,0032 | Reactome |
| Downstream signaling of activated FGFR3 | 267 | 10 | 3,80% | 0,00139 | 0,0032 | Reactome |
| Downstream signaling of activated FGFR4 | 267 | 10 | 3,80% | 0,00139 | 0,0032 | Reactome |
| Tachykinin receptors bind tachykinins | 5 | 2 | 40,00% | 0,00141 | 0,00324 | Reactome |
| Wnt | 70 | 5 | 7,20% | 0,00142 | 0,00325 | NetPath |
| role of mal in rho-mediated activation of srf | 19 | 3 | 15,80% | 0,00144 | 0,00325 | BioCarta |
| Bladder cancer - Homo sapiens (human) | 41 | 4 | 9,80% | 0,00144 | 0,00325 | KEGG |
| Degradation of beta catenin | 19 | 3 | 15,80% | 0,00144 | 0,00325 | PID |
| S1P1 pathway | 19 | 3 | 15,80% | 0,00144 | 0,00325 | PID |
| CD209 (DC-SIGN) signaling | 19 | 3 | 15,80% | 0,00144 | 0,00325 | Reactome |
| Platelet calcium homeostasis | 19 | 3 | 15,80% | 0,00144 | 0,00325 | Reactome |
| Insulin signaling pathway - Homo sapiens (human) | 140 | 7 | 5,00% | 0,00149 | 0,00335 | KEGG |
| Signaling by FGFR3 | 270 | 10 | 3,70% | 0,00151 | 0,00337 | Reactome |
| Signaling by FGFR4 | 270 | 10 | 3,70% | 0,00151 | 0,00337 | Reactome |
| Alpha6Beta4Integrin | 71 | 5 | 7,10% | 0,00152 | 0,00339 | NetPath |
| Signaling by FGFR1 | 271 | 10 | 3,70% | 0,00155 | 0,00345 | Reactome |
| CLEC7A (Dectin-1) signaling | 42 | 4 | 9,50% | 0,00158 | 0,0035 | Reactome |
| Inositol phosphate metabolism - Homo sapiens (human) | 71 | 5 | 7,00% | 0,00162 | 0,00357 | KEGG |
| KitReceptor | 71 | 5 | 7,00% | 0,00162 | 0,00357 | NetPath |
| Signaling by FGFR2 | 273 | 10 | 3,70% | 0,00164 | 0,0036 | Reactome |
| EGF-Core | 105 | 6 | 5,70% | 0,00165 | 0,00363 | Signalink |
| egf signaling pathway | 20 | 3 | 15,00% | 0,00167 | 0,00365 | BioCarta |
| Canonical Wnt signaling pathway | 20 | 3 | 15,00% | 0,00167 | 0,00365 | PID |
| Signaling by FGFR | 274 | 10 | 3,70% | 0,00168 | 0,00366 | Reactome |
| Signaling by ERBB2 | 277 | 10 | 3,60% | 0,00182 | 0,00396 | Reactome |
| Hepatitis B - Homo sapiens (human) | 146 | 7 | 4,80% | 0,0019 | 0,0041 | KEGG |
| Downstream signal transduction | 279 | 10 | 3,60% | 0,00192 | 0,00415 | Reactome |
| igf-1 signaling pathway | 21 | 3 | 14,30% | 0,00194 | 0,00415 | BioCarta |
| NGF | 21 | 3 | 14,30% | 0,00194 | 0,00415 | INOH |
| SHC-mediated cascade:FGFR1 | 21 | 3 | 14,30% | 0,00194 | 0,00415 | Reactome |
| Carbohydrate digestion and absorption - Homo sapiens (human) | 45 | 4 | 8,90% | 0,00204 | 0,00434 | KEGG |
| a6b1 and a6b4 Integrin signaling | 45 | 4 | 8,90% | 0,00204 | 0,00434 | PID |
| Insulin Pathway | 45 | 4 | 8,90% | 0,00204 | 0,00434 | PID |
| Focal Adhesion | 190 | 8 | 4,20% | 0,00208 | 0,00439 | Wikipathways |
| Leptin signaling pathway | 76 | 5 | 6,60% | 0,00219 | 0,00461 | Wikipathways |
| Prolactin Signaling Pathway | 76 | 5 | 6,60% | 0,00219 | 0,00461 | Wikipathways |
| inhibition of cellular proliferation by gleevec | 22 | 3 | 13,60% | 0,00222 | 0,00461 | BioCarta |
| ras-independent pathway in nk cell-mediated cytotoxicity | 22 | 3 | 13,60% | 0,00222 | 0,00461 | BioCarta |
| Inositol phosphate metabolism | 46 | 4 | 8,70% | 0,00222 | 0,00461 | Reactome |
| Joubert syndrome | 22 | 3 | 13,60% | 0,00222 | 0,00461 | SMPDB |
| Phosphatidylinositol Phosphate Metabolism | 22 | 3 | 13,60% | 0,00222 | 0,00461 | SMPDB |
| Aryl Hydrocarbon Receptor | 46 | 4 | 8,70% | 0,00222 | 0,00461 | Wikipathways |
| EPH-Ephrin signaling | 77 | 5 | 6,50% | 0,00232 | 0,00479 | Reactome |
| insulin | 78 | 5 | 6,40% | 0,00245 | 0,00506 | INOH |
| how progesterone initiates the oocyte maturation | 23 | 3 | 13,00% | 0,00253 | 0,00517 | BioCarta |
| tumor suppressor arf inhibits ribosomal biogenesis | 24 | 3 | 13,00% | 0,00253 | 0,00517 | BioCarta |
| Regulation of RAS by GAPs | 24 | 3 | 13,00% | 0,00253 | 0,00517 | Reactome |
| SHC-mediated cascade:FGFR4 | 23 | 3 | 13,00% | 0,00253 | 0,00517 | Reactome |
| Platelet homeostasis | 79 | 5 | 6,30% | 0,00259 | 0,00528 | Reactome |
| VEGFR2 mediated cell proliferation | 198 | 8 | 4,10% | 0,0026 | 0,00528 | Reactome |
| RHO GTPases Activate Formins | 115 | 6 | 5,20% | 0,00262 | 0,00531 | Reactome |
| Signaling by EGFR | 292 | 10 | 3,40% | 0,00268 | 0,00542 | Reactome |
| Physiological and Pathological Hypertrophy of the Heart | 24 | 3 | 12,50% | 0,00287 | 0,00578 | Wikipathways |
| Leukocyte transendothelial migration - Homo sapiens (human) | 118 | 6 | 5,10% | 0,00298 | 0,00599 | KEGG |
| PI3K-Akt signaling pathway - Homo sapiens (human) | 347 | 11 | 3,20% | 0,00303 | 0,00608 | KEGG |
| insulin Mam | 82 | 5 | 6,10% | 0,00305 | 0,0061 | INOH |
| tpo signaling pathway | 25 | 3 | 12,00% | 0,00323 | 0,00637 | BioCarta |
| update your name in edit mode | 25 | 3 | 12,00% | 0,00323 | 0,00637 | PharmGKB |
| Vemurafenib Pathway, Pharmacodynamics | 25 | 3 | 12,00% | 0,00323 | 0,00637 | PharmGKB |
| Signal Transduction of S1P Receptor | 25 | 3 | 12,00% | 0,00323 | 0,00637 | Wikipathways |
| Antigen activates B Cell Receptor (BCR) leading to generation of second messengers | 55 | 4 | 7,80% | 0,00324 | 0,00637 | Reactome |
| PI Metabolism | 51 | 4 | 7,80% | 0,00324 | 0,00637 | Reactome |
| Downstream signaling events of B Cell Receptor (BCR) | 120 | 6 | 5,00% | 0,00324 | 0,00637 | Reactome |
| Signaling by Rho GTPases | 404 | 12 | 3,00% | 0,00331 | 0,00648 | Reactome |
| Signaling by PDGF | 301 | 10 | 3,30% | 0,00334 | 0,00653 | Reactome |
| Focal adhesion - Homo sapiens (human) | 207 | 8 | 3,90% | 0,00352 | 0,00687 | KEGG |
| signaling pathway from g-protein families | 26 | 3 | 11,50% | 0,00362 | 0,00703 | BioCarta |
| SHC-mediated cascade:FGFR2 | 26 | 3 | 11,50% | 0,00362 | 0,00703 | Reactome |
| t cell receptor signaling pathway | 55 | 4 | 7,50% | 0,00373 | 0,00718 | BioCarta |
| Signaling events mediated by PTP1B | 53 | 4 | 7,50% | 0,00373 | 0,00718 | PID |
| TCR signaling in naïve CD8+ T cells | 55 | 4 | 7,50% | 0,00373 | 0,00718 | PID |
| Integrin | 124 | 6 | 4,80% | 0,00381 | 0,00733 | INOH |
| Rapid glucocorticoid signaling | 8 | 2 | 25,00% | 0,00384 | 0,00733 | PID |
| Axonal growth inhibition (RHOA activation) | 9 | 2 | 25,00% | 0,00384 | 0,00733 | Reactome |
| Sema4D mediated inhibition of cell attachment and migration | 8 | 2 | 25,00% | 0,00384 | 0,00733 | Reactome |
| ErbB Signaling Pathway | 55 | 4 | 7,40% | 0,00399 | 0,00759 | Wikipathways |
| pdgf signaling pathway | 27 | 3 | 11,10% | 0,00404 | 0,00765 | BioCarta |
| role of erk5 in neuronal survival pathway | 27 | 3 | 11,10% | 0,00404 | 0,00765 | BioCarta |
| HTLV-I infection - Homo sapiens (human) | 259 | 9 | 3,50% | 0,00405 | 0,00765 | KEGG |
| Signaling by SCF-KIT | 264 | 9 | 3,40% | 0,00447 | 0,0084 | Reactome |
| role of erbb2 in signal transduction and oncology | 28 | 3 | 10,70% | 0,00449 | 0,0084 | BioCarta |
| EGF | 28 | 3 | 10,70% | 0,00449 | 0,0084 | INOH |
| Anti-diabetic Drug Potassium Channel Inhibitors Pathway, Pharmacodynamics | 28 | 3 | 10,70% | 0,00449 | 0,0084 | PharmGKB |
| Phosphatidylinositol phosphate metabolism | 91 | 5 | 5,50% | 0,00477 | 0,00891 | EHMN |
| Regulation of lipolysis in adipocytes - Homo sapiens (human) | 57 | 4 | 7,00% | 0,00485 | 0,00903 | KEGG |
| Formyl peptide receptors bind formyl peptides and many other ligands | 9 | 2 | 22,20% | 0,0049 | 0,00909 | Reactome |
| p75NTR regulates axonogenesis | 10 | 2 | 22,20% | 0,0049 | 0,00909 | Reactome |
| fc epsilon receptor i signaling in mast cells | 29 | 3 | 10,30% | 0,00496 | 0,00914 | BioCarta |
| Thyroid cancer - Homo sapiens (human) | 29 | 3 | 10,30% | 0,00496 | 0,00914 | KEGG |
| disassembly of the destruction complex and recruitment of AXIN to the membrane | 29 | 3 | 10,30% | 0,00496 | 0,00914 | Reactome |
| Fc gamma R-mediated phagocytosis - Homo sapiens (human) | 92 | 5 | 5,40% | 0,005 | 0,00919 | KEGG |
| Hepatitis C - Homo sapiens (human) | 133 | 6 | 4,50% | 0,00536 | 0,00983 | KEGG |
| IGF1 pathway | 30 | 3 | 10,00% | 0,00546 | 0,00999 | PID |
| FoxO signaling pathway - Homo sapiens (human) | 134 | 6 | 4,50% | 0,00556 | 0,0101 | KEGG |
| regulation of splicing through sam68 | 10 | 2 | 20,00% | 0,00608 | 0,011 | BioCarta |
| GP1b-IX-V activation signalling | 10 | 2 | 20,00% | 0,00608 | 0,011 | Reactome |
| CD4 T cell receptor signaling-NFkB cascade | 97 | 5 | 5,20% | 0,00625 | 0,0113 | INOH |
| rho cell motility signaling pathway | 33 | 3 | 9,40% | 0,00656 | 0,0118 | BioCarta |
| Syndecan-4-mediated signaling events | 32 | 3 | 9,40% | 0,00656 | 0,0118 | PID |
| integrin signaling pathway | 33 | 3 | 9,10% | 0,00715 | 0,0128 | BioCarta |
| EPHA forward signaling | 33 | 3 | 9,10% | 0,00715 | 0,0128 | PID |
| Trk receptor signaling mediated by the MAPK pathway | 33 | 3 | 9,10% | 0,00715 | 0,0128 | PID |
| Endothelin Pathways | 33 | 3 | 9,10% | 0,00715 | 0,0128 | Wikipathways |
| RHO GTPases Activate NADPH Oxidases | 11 | 2 | 18,20% | 0,00737 | 0,0131 | Reactome |
| Oncostatin M Signaling Pathway | 65 | 4 | 6,20% | 0,00772 | 0,0137 | Wikipathways |
| CD40/CD40L signaling | 34 | 3 | 8,80% | 0,00778 | 0,0137 | PID |
| DAG and IP3 signaling | 34 | 3 | 8,80% | 0,00778 | 0,0137 | Reactome |
| Signaling of Hepatocyte Growth Factor Receptor | 34 | 3 | 8,80% | 0,00778 | 0,0137 | Wikipathways |
| CRH | 35 | 3 | 8,60% | 0,00843 | 0,0148 | NetPath |
| FAS (CD95) signaling pathway | 35 | 3 | 8,60% | 0,00843 | 0,0148 | PID |
| Glucagon-like Peptide-1 (GLP1) regulates insulin secretion | 35 | 3 | 8,60% | 0,00843 | 0,0148 | Reactome |
| TCR signaling in naïve CD4+ T cells | 69 | 4 | 6,00% | 0,00858 | 0,015 | PID |
| map kinase inactivation of smrt corepressor | 12 | 2 | 16,70% | 0,00878 | 0,0153 | BioCarta |
| Interleukin receptor SHC signaling | 195 | 7 | 3,60% | 0,00897 | 0,0156 | Reactome |
| BCR signaling pathway | 68 | 4 | 5,90% | 0,00903 | 0,0157 | PID |
| Signaling events mediated by VEGFR1 and VEGFR2 | 68 | 4 | 5,90% | 0,00903 | 0,0157 | PID |
| EGFR interacts with phospholipase C-gamma | 36 | 3 | 8,30% | 0,00912 | 0,0158 | Reactome |
| PLC-gamma1 signalling | 36 | 3 | 8,30% | 0,00912 | 0,0158 | Reactome |
| MicroRNAs in cancer - Homo sapiens (human) | 297 | 6 | 4,00% | 0,00948 | 0,0164 | KEGG |
| IL2 signaling events mediated by PI3K | 37 | 3 | 8,10% | 0,00984 | 0,0168 | PID |
| Netrin-1 signaling | 37 | 3 | 8,10% | 0,00984 | 0,0168 | Reactome |
| Platelet Aggregation (Plug Formation) | 37 | 3 | 8,10% | 0,00984 | 0,0168 | Reactome |
| PLCG1 events in ERBB2 signaling | 37 | 3 | 8,10% | 0,00984 | 0,0168 | Reactome |
| p75(NTR)-mediated signaling | 70 | 4 | 5,70% | 0,00998 | 0,017 | PID |
