# Supplementary material for: Mechanisms of action of sacubitril/valsartan on cardiac remodeling: a systems biology approach
Source: NPJ Syst Biol Appl. 2017 Apr 18;3:12. doi: 10.1038/s41540-017-0013-4 (PMC5460292; doi:10.1038/s41540-017-0013-4)
Supplement: Supplementary file 2 — Supplementary Figure Legends [file 41540_2017_13_MOESM2_ESM.docx]

Supplementary Figure 1: Gene/Protein restrictions selection. First, those proteins in the microarray data that fulfilled and passed the filters after translating the pig proteome were used to further analysis. Then, the proteins are tagged accordingly to their state, activated or inhibited, in healthy conditions. Using this information as baseline, we restrict the models to account only for those proteins that vary from these values on the follow-up. Red circles refer to human data obtained. Blue circles refer to pig data obtained. Grated circles depict contradictory or non-homologous information. Green arrows depict an example of activated proteins. Red arrows depict an example of inhibit proteins. Black crosses represent the process of discarding those proteins that does not differ from baseline conditions, which have not been used for further analysis.

Supplementary Figure 2: Biological Effectors Database (BED) representation. BED is a database property of Anaxomics Biotech SL. It includes several conditions (in our case we focused on MI and HF), the motives that cause them and the effector proteins/genes related to those conditions.

**Supplementary Figure 3: Artificial neuronal networks (ANN) depiction.** The learning methodology used consisted in a mixture of neural networks as a model, trained with a gradient descent algorithm to approximate the values of the given truth-table. Known input (Blue circles) refers to any element that affects gene/protein expression level or activity in the network. Every link has been previously described in the literature. TPMS technology collect these stimulus, relates them with the known output (in this case adverse cardiac remodeling; red circle) and traces back the pathway most likely to be responsible for the observed outcome (Green circles refers to our pool of proteins/genes).

**Supplementary Figure 4: TPMS technology illustration.** TPMS Technology takes into account all known stimulus (inputs) that may affect our group of proteins (136 proteins in this case) under the pathology of study. Then, allows us to trace back the molecular pathway most likely to cause the known response (output) (in this case adverse cardiac remodeling). Colored nodes on the left circle represent different proteins on basal condition that are affected by different stimulus. Colored nodes on the right circle represent the same proteins responding to the stimulus; hence the change of color on some examples. MoA= Mechanism of Action; AEs= drugs Adverse Effects.

**Supplementary Figure 5: Valsartan’s effects on cardiac remodeling.** Highlights extracted from the MoA (Figure 2) that explain which proteins affected by Valsartan are ultimately involved in reducing adverse cardiac remodeling via specific motives (e.g. Hypertrophy), according to the mathematical models.

**Supplementary Figure 6: Representation of the mechanistic roles of an alternative synergistic pathway.** The model indicates a second potential synergistic pathway for Sacubitril/Valsartan’s effects on cardiac remodeling. Red lines indicate inhibition, green lines indicate activation, and dashed blue lines indicate an indirect relationship. Grey dotted-line circles encompass the proteins affected either by Sacubitril or Valsartan.

**Supplementary Figure 7: Sacubitril’s effects on cardiac remodeling.** Highlights extracted from the MoA (Figure 2) that explain which proteins affected by Sacubitril are ultimately involved in reducing adverse cardiac remodeling via specific motives (e.g. Hypertrophy), according to the mathematical models.
