# Supplementary figures and images for: Mechanisms of action of sacubitril/valsartan on cardiac remodeling: a systems biology approach
Source: NPJ Syst Biol Appl. 2017 Apr 18;3:12. doi: 10.1038/s41540-017-0013-4 (PMC5460292; doi:10.1038/s41540-017-0013-4)

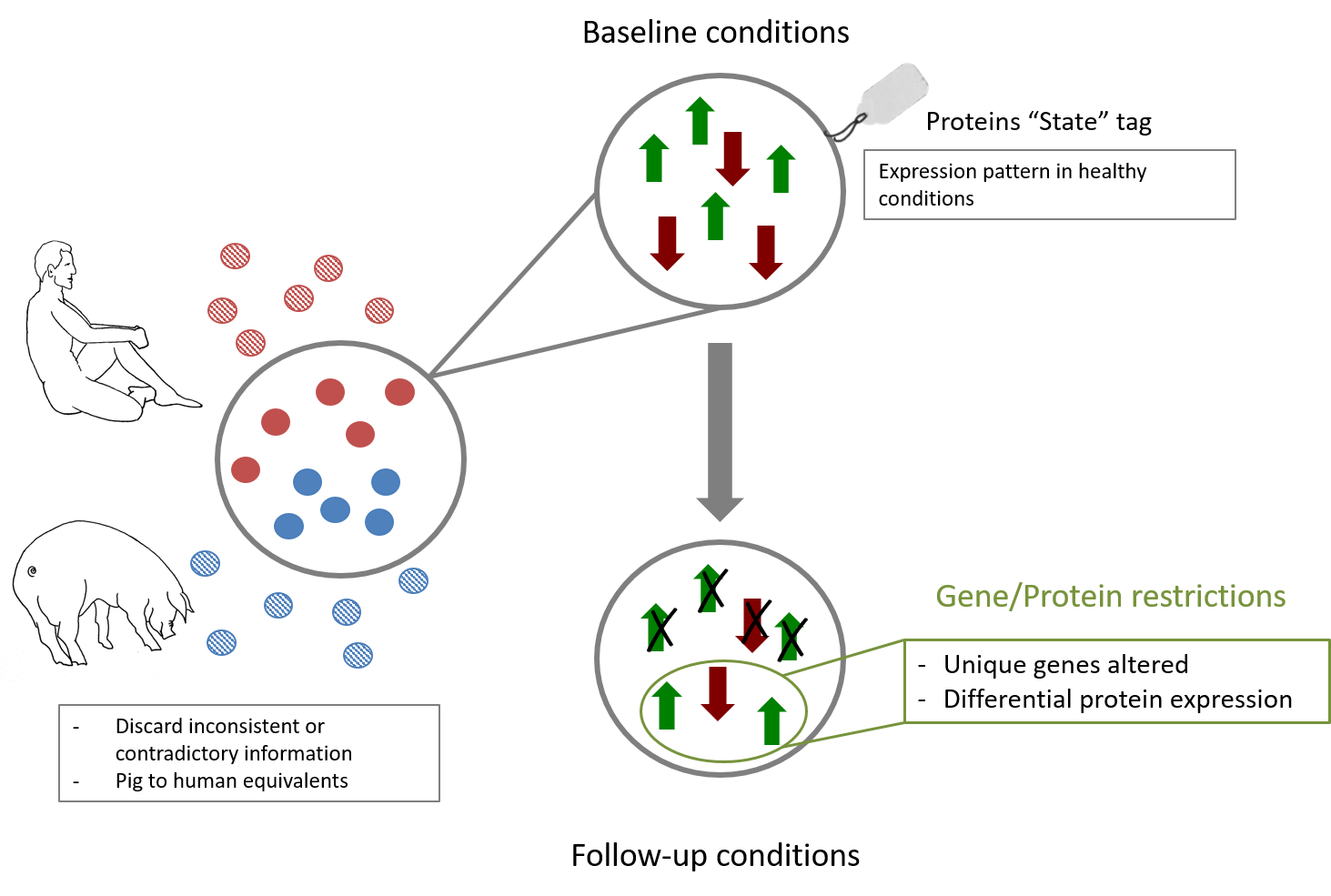

Supplement: Supplementary file 3 — Supplementary Figure 1 [file 41540_2017_13_MOESM3_ESM.tif]

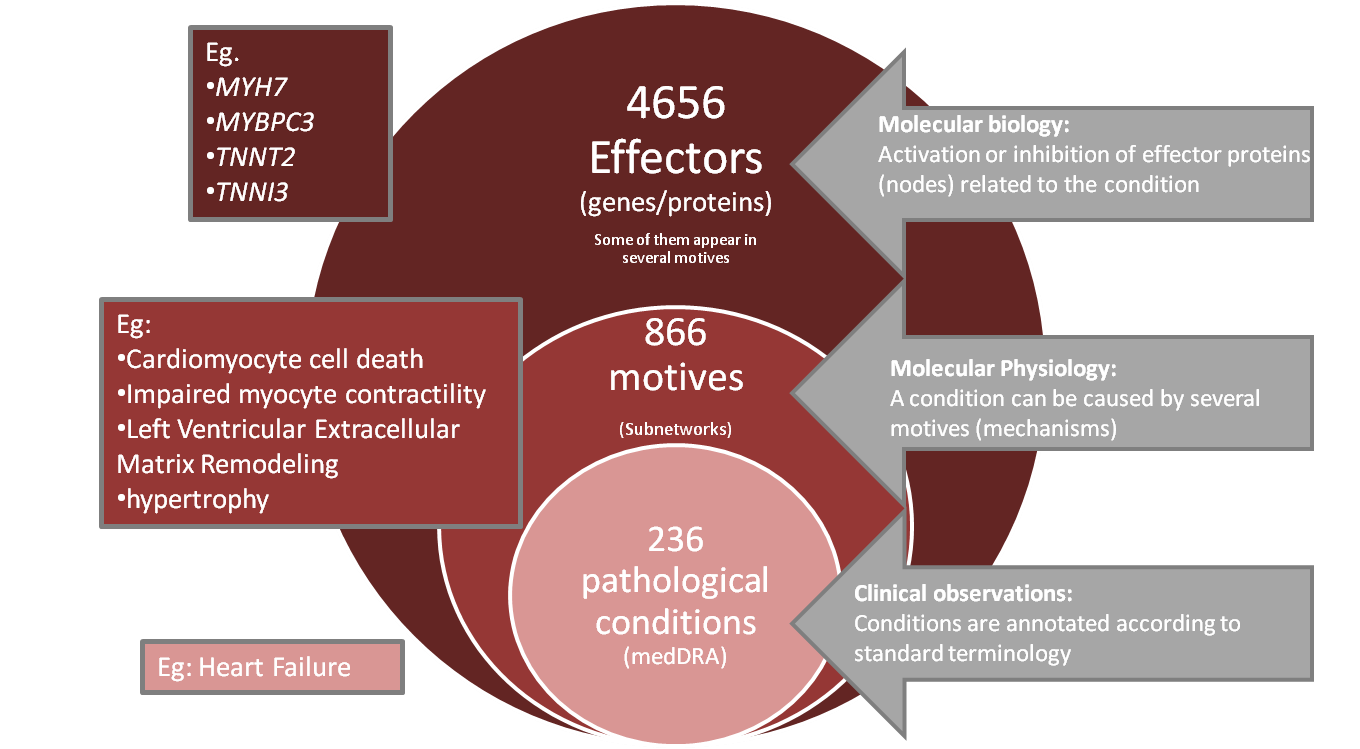

Supplement: Supplementary file 4 — Supplementary Figure 2 [file 41540_2017_13_MOESM4_ESM.tif]

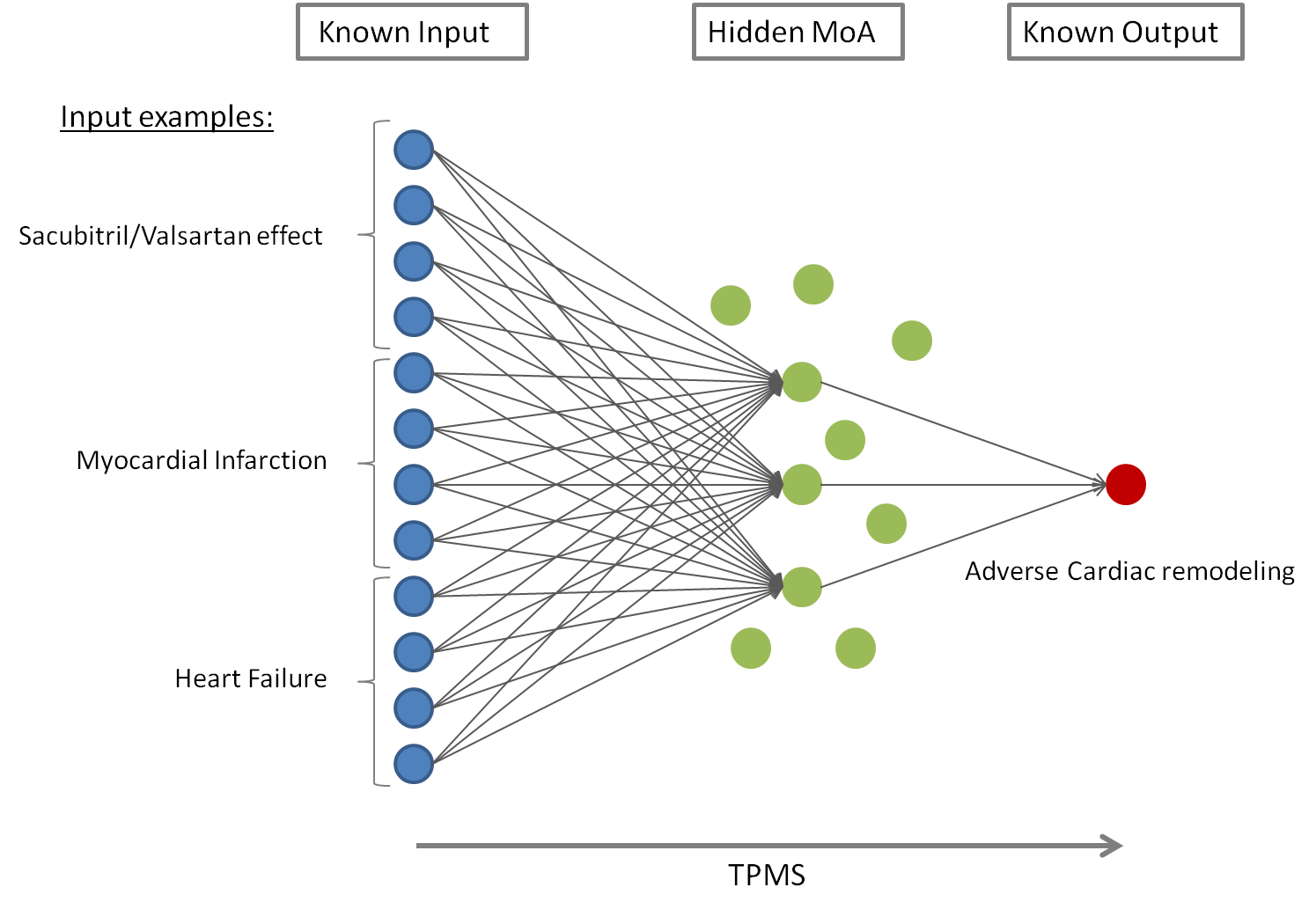

Supplement: Supplementary file 5 — Supplementary Figure 3 [file 41540_2017_13_MOESM5_ESM.tif]

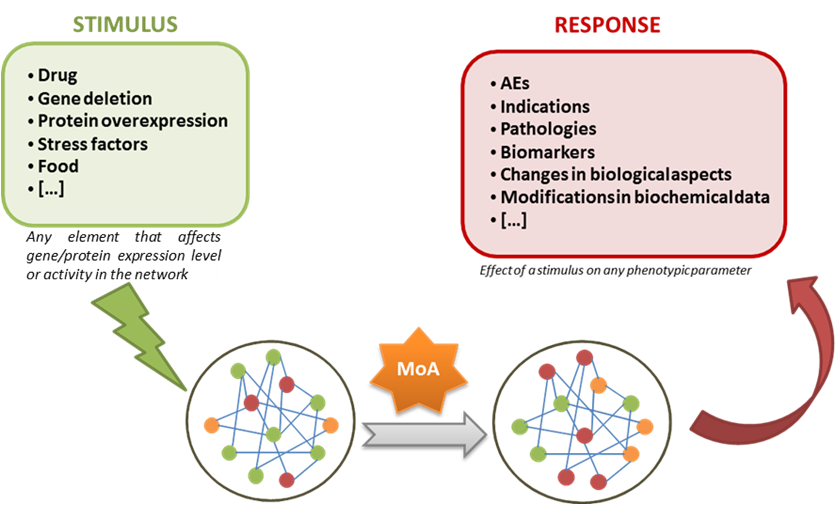

Supplement: Supplementary file 6 — Supplementary Figure 4 [file 41540_2017_13_MOESM6_ESM.tif]

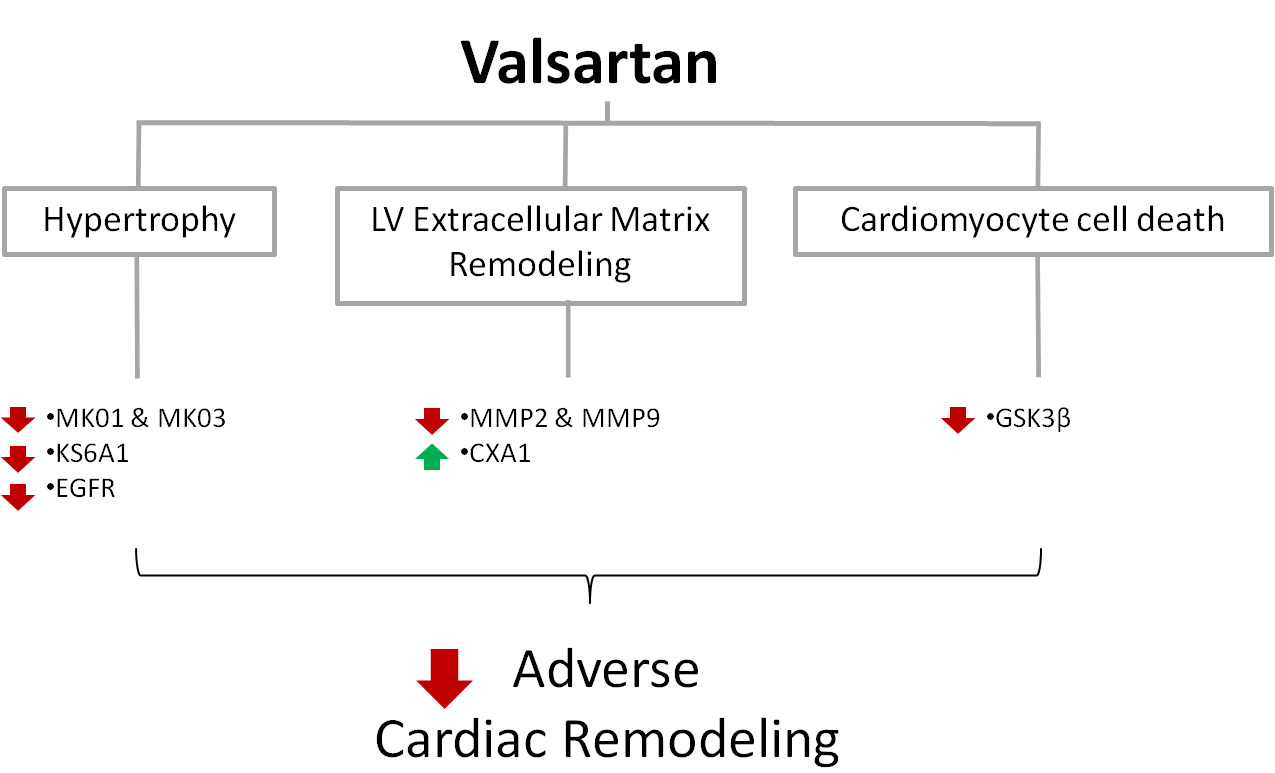

Supplement: Supplementary file 7 — Supplementary Figure 5 [file 41540_2017_13_MOESM7_ESM.tif]

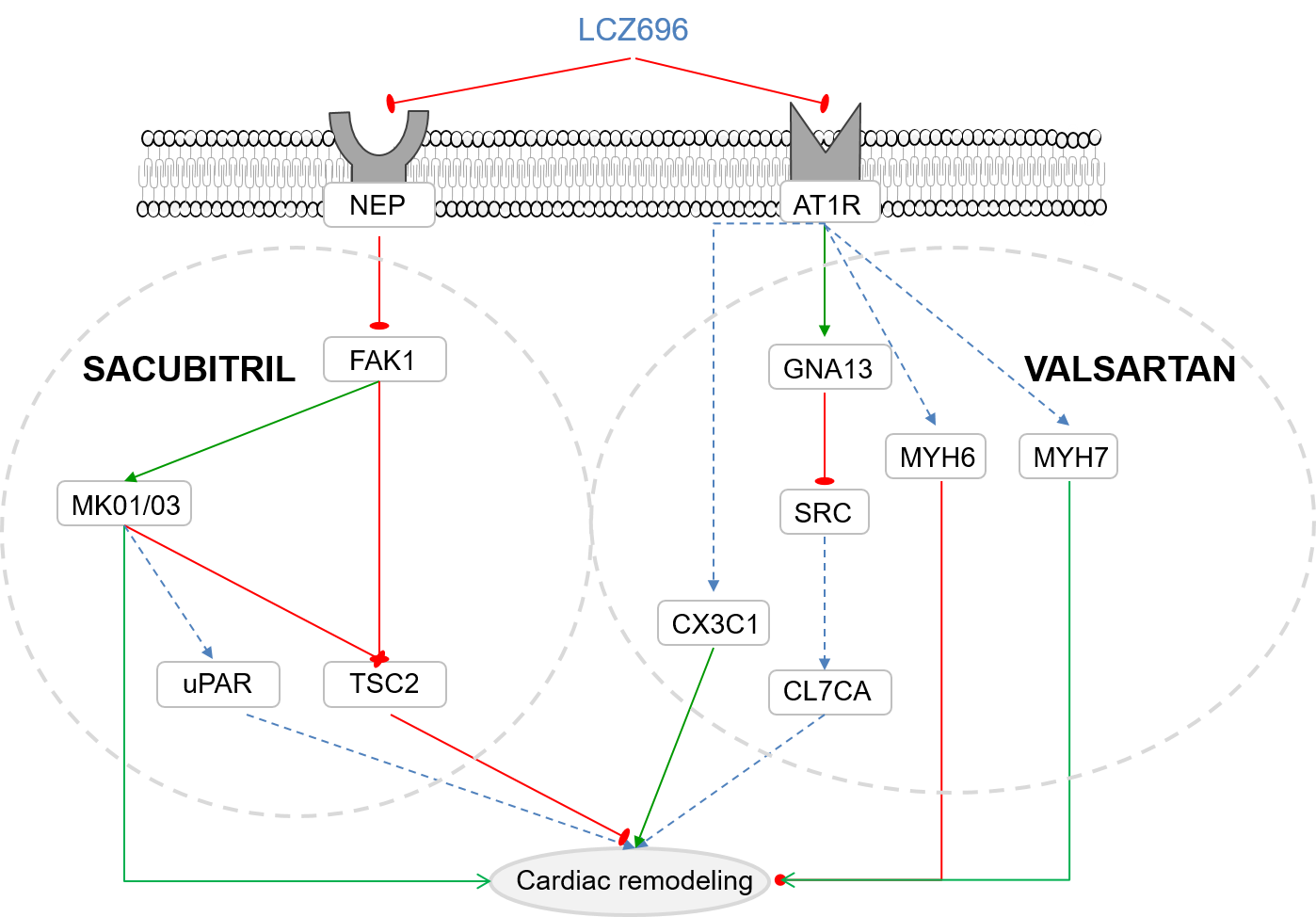

Supplement: Supplementary file 8 — Supplementary Figure 6 [file 41540_2017_13_MOESM8_ESM.tif]

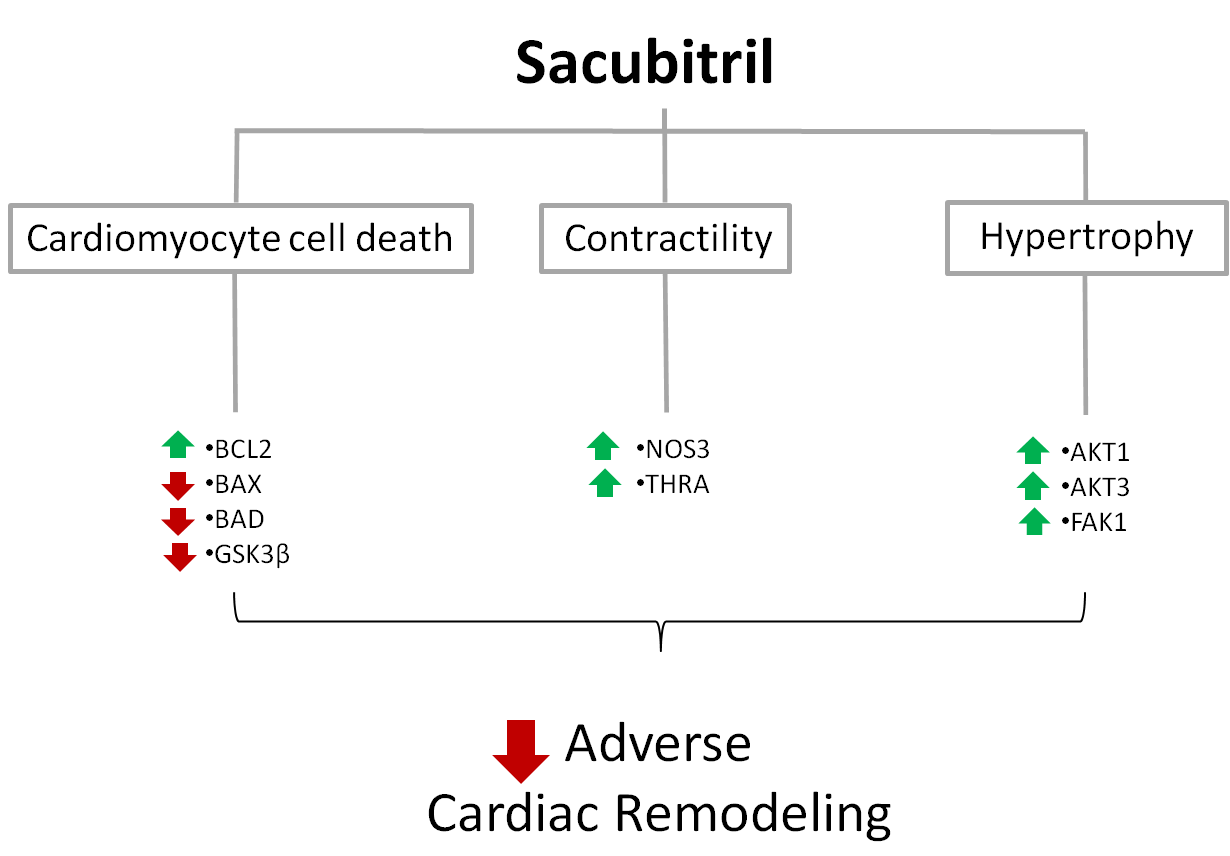

Supplement: Supplementary file 9 — Supplementary Figure 7 [file 41540_2017_13_MOESM9_ESM.tif]
